# Snake Venomics: Fundamentals, Recent Updates, and a Look to the Next Decade

**DOI:** 10.3390/toxins14040247

**Published:** 2022-03-30

**Authors:** Choo Hock Tan

**Affiliations:** 1 Venom Research and Toxicology Laboratory, Department of Pharmacology, Faculty of Medicine, University of Malaya, Kuala Lumpur 50603, Malaysia; tanch@um.edu.my; 2 Department of Life Science, Institute of Bioinformatics and Structural Biology, National Tsing Hua University, Hsinchu 30013, Taiwan

**Keywords:** venom, toxin, protein decomplexation, next-generation sequencing, proteomics, transcriptomics, genomics

## Abstract

Venomic research, powered by techniques adapted from proteomics, transcriptomics, and genomics, seeks to unravel the diversity and complexity of venom through which knowledge can be applied in the treatment of envenoming, biodiscovery, and conservation. Snake venom proteomics is most extensively studied, but the methods varied widely, creating a massive amount of information which complicates data comparison and interpretation. Advancement in mass spectrometry technology, accompanied by growing databases and sophisticated bioinformatic tools, has overcome earlier limitations of protein identification. The progress, however, remains challenged by limited accessibility to samples, non-standardized quantitative methods, and biased interpretation of -omic data. Next-generation sequencing (NGS) technologies enable high-throughput venom-gland transcriptomics and genomics, complementing venom proteomics by providing deeper insights into the structural diversity, differential expression, regulation and functional interaction of the toxin genes. Venomic tissue sampling is, however, difficult due to strict regulations on wildlife use and transfer of biological materials in some countries. Limited resources for techniques and funding are among other pertinent issues that impede the progress of venomics, particularly in less developed regions and for neglected species. Genuine collaboration between international researchers, due recognition of regional experts by global organizations (e.g., WHO), and improved distribution of research support, should be embraced.

## 1. Venom: What’s in Thy Name?

“The beginning of right wisdom (politics) is to call things (people) by their right names.”—This saying from *The Analects of Confucius* perhaps justifies humans’ compulsion to name things for orderliness. In biology, Nature itself is forever messy (though elegant), and science is merely tentative as concepts, ideas, and nomenclatures/definitions continue to be revised. Thus, when discussing *venomics*, we first ought to ask, *what is venom?* Then we should learn to know more: *Where does it come from? What is venom for? How does it work?* Fundamental questions like these, nevertheless, easily spark critical debate among scientists (toxinologists, to be exact). To our benefit, there is a fairly widespread consensus nowadays regarding the definition of venom: It is a biological secretion produced by specialized cells or tissues in a venomous animal, stored in the cells or glandular lumens, and actively delivered into another animal by inflicting a wound (no matter how small) through a specific suite of behavior for predation, diet, defense, or other ecological interactions [1,2].

Now one should be able to quickly discern between venom and poison—the latter is traditionally regarded as “any kind of toxic substance”, which could be natural or synthetic, actively or passively delivered, and has an existence not confined to the Animal Kingdom. Venomous animals are widespread; they are distributed across various phyla, from miniature invertebrates, such as the Irukanji jellyfish, to mammals, such as the platypus. The functional phenotype of being able to de novo synthesize and utilize venom to the success of survival is an evolutionarily defining adaptive trait. For many predatory animals, venom is indispensable for hunting and digestion of prey. In others, venom is used for defense against predators, typically by inducing pain. Also, venom plays important roles in some ecological interactions, such as intraspecific competition (e.g., platypus) [3], conspecific communication (e.g., wasps) [4], chemical detoxification (e.g., formicine ants) [5], and detection of ambushed then envenomed prey (e.g., rattlesnakes) [6]. Of the many different venomous taxa, snakes and their venoms are perhaps the most extensively studied for their medical importance in two somewhat contradictory aspects: (1) Snakebite—a deadly interaction vexing humans for millennia that is still unresolved [7,8]; (2) Bioprospecting potential—venom contains myriad pharmacologically active components (toxins) that are naturally chiseled against the mammalian physiology, serving as leads for drug discovery [9,10].

The development of venomous phenotypes facilitated the shift from a mechanical to a more efficient biochemical method of predation in snakes (order: Serpentes), and is believed to be responsible for their rapid diversification and enormous expansion across the globe [11]. The evolutionary history of snake venom is, however, debatable and unresolved. Prior to 2014, the dominant view was that the reptilian venoms originated just once circa 170 million years ago within a clade named “Toxicofera”, proposed based on the presence of similar venom proteins by nuclear gene sequencing, and homology of venom-delivery system in a number of lizards and snakes [12,13]. Under the *toxicoferan (single-origin) hypothesis*, the original toxicoferan venom was assembled in a pair of oral glands and subsequently diversified in various lineages including Serpentes, Anguimorpha, and Iguania. The toxicoferans then evolved independently along with their means of injecting venom into prey, such as the evolution of a front-fanged venom-delivery system from the ancestral rear-fanged system. The *single-origin hypothesis* suggests that the mechanism of evolution in most cases has been gene duplication followed by natural selection for adaptive traits [14]. The various means of venom adaptation created a considerable debate about the definition of venom and venomous snakes [15,16]. Subsequent studies demonstrated that many of the so-called venom protein genes had highly expressed homologs in other bodily tissues of non-venomous snakes, e.g., the Burmese python [17,18], suggesting that pythons represented a period in snake evolution before major venom development, whereas most venom evolution took place after the venomous caenophidian or colubroidian snakes diverged from other snakes, accompanied with an expansion of venom gene families. This school of thought (*independent origin hypothesis*) holds the view that snake venoms had evolved more than once, independently, in numerous lineages.

## 2. Venomics: The Unravelling Tool

Regardless of the debatable venom origin, snake venom aptly illustrates the composition complexity of toxins shaped by selection pressure and reflects the functional adaptions of snakes to diverse niches. As complex mixtures of bioactive proteins and peptides (collectively called toxins), snake venoms tend to be inherently variable within and between species [19,20,21]. Medically motivated snake venom research has since mainly targeted the following aspects: (a) the genetic, biochemical, and physicochemical properties of snake venoms; (b) the mechanisms of action and potential uses therefrom; (c) the management of snakebite envenoming as in developing diagnostics, antidotes including antivenom, and strategic protocol of treatment. What lies in the core knowledge body is the details of venom composition that must be demonstratable through gene and protein profiling to allow a deeper quest into the structures, activities, functionality, and application of the toxins [22,23].

**Figure 1:**
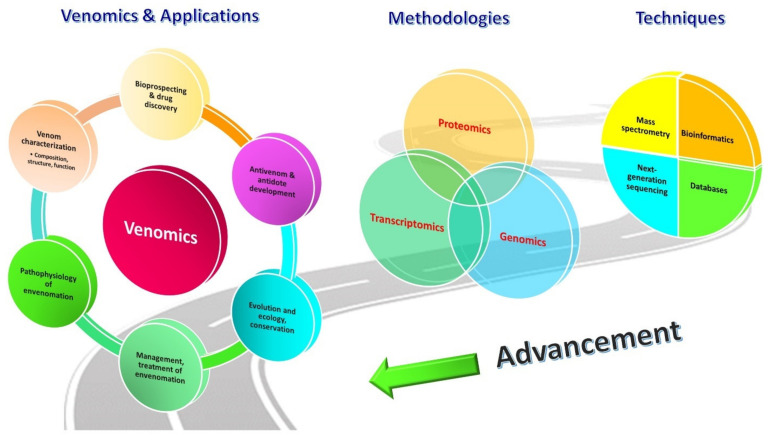
Venomics: Advancing proteomic, transcriptomic, and genomic platforms, supported by high-throughput sequencing techniques for protein/peptide, RNA and DNA, growing databases, knowledge-bases and bio-computing algorithms, which drive the advancement of venomics. Venomics contributes toward the knowledge of venom evolution, toxin functionality, pathophysiology, and treatment of envenomation, and paves the way for biodiscovery, as well as improvement of antivenom production.

In this regard, the advent of OMICS technology has completely revolutionized the study of venom composition. Dr. Juan J. Calvete from the Instituto de Biomedicina de Valencia pioneered the field, and the term “venomics” has since been coined to denote the global profiling of venom by means of proteomics (on the secreted product), transcriptomics (on venom-secreting tissue or organ, e.g., venom gland) and genomics (on any body tissue), coupled with bioinformatic studies [24,25,26]. At present, venomics is used quite commonly in the field to represent “proteomics of venom” to the extent that both are applied almost interchangeably. Venomics earns its glamor and popularity for a reason: Prior to the venomic era, bioassay-guided protein purification was the only platform to identify and characterize protein components of interest in snake venom; hence, comprehensive profiling of global proteins and genes, and the elucidation of their dynamic crosstalk were just unrealistic back then. The venomic strategy opens a totally new chapter into the pursuit of this knowledge. Readily supported by new sequencing techniques for protein/peptide, RNA and DNA, as well as the rapidly expanding databases, knowledge-bases and bio-computing algorithms, venomics allows high throughput comprehensive study that yields enormous data for a venom’s global constitution, even for minor components that exist in a very low quantity in the sample [27,28]. This revolutionizing breakthrough by venomics has propelled tremendous growth of the knowledge body on various aspects, including venom evolution, toxin functionality, pathophysiology and treatment of envenomation, antivenom production, and biodiscovery (Figure 1) [29,30,31].

## 3. Strategies in Venomics: No ‘One-Size-Fits-All’

**Figure 2:**
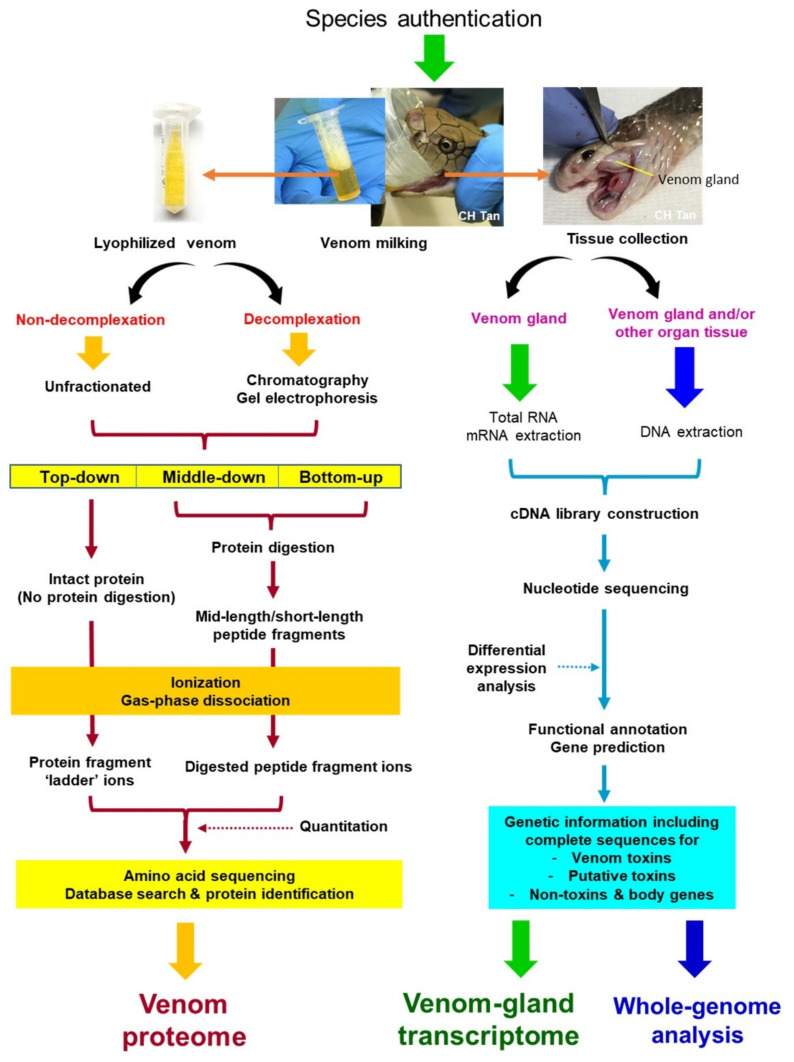
Venomic workflow incorporating proteomics, transcriptomics, and genomics. Proteomics utilizes venom (proteins) and adopts various profiling approaches, which can be briefly classified into decomplexation (involving venom fractionation by chromatography and gel electrophoresis) and non-decomplexation strategies (using unfractionated whole venom), followed by amino acid sequencing applying mass spectrometry. Bottom-up proteomics is the conventional and most commonly used technique, whereas top-down (and middle-down) sequencing are emerging methods that offer new insights in recent venomics. Transcriptomics and genomics require tissue samples from the venomous animals for RNA/DNA extraction. Next-generation sequencing (NGS) of nucleotides is a massively parallel sequencing technology that offers ultra-high throughput, scalability, and speed for transcriptome and genome assembly.

The progression of venomics, since its inception, has always been reliant on and limited by the advancement of technology, which is fast evolving. In every few other years, a number of reviews will be published comparing the different venomic workflows, in particular on snake venoms [25,32,33,34]. Ostensibly, a single analytical method is insufficient to unravel the complexity of living systems, and each approach has its strengths and limitations. The technical and conceptual frameworks of venomics advanced and diversified over time with increasing flexibility, which allows the methodology to mold and fit according to the sample conditions, research objectives, and resources available at a time. The conceptual framework of current venomics, incorporating proteomics, transcriptomics, and genomics, is illustrated in Figure 2. Recent modification and variation of methods and techniques are incorporated in the overview depicted by snake venomics.

### 3.1. Proteomics of Snake Venom

Proteomics of snake venom is by far the most popular and common study in venomics. The process begins with venom collection, a simple but critical step which has tremendous impact on the downstream analysis and data interpretation. Manual “milking” of venom is by far the most common method employed for venom extraction from living venomous animals including snakes (Figure 1) and arthropods [29,35,36], in contrast to venom obtained by surgical extraction from dissected tissues, such as in cnidarian jellyfishes [37]. The venom collected can be directly stored in −20 °C or snap-frozen at −80 °C, while most researchers prefer to lyophilize or freeze-dry the samples for long-term stability of the contents. The sample quality control, traceability, and standard operating procedures for reproducibility of study should be emphasized throughout the process of venom collection. The specimen must be correctly authenticated (*viz*, the exact species) and the extent of sampling must be determined (*viz*, the number, sex, and geographical origin of the specimen) where possible to ensure the validity of species, the representativeness of specimen, and data reproducibility.

#### 3.1.1. Evolutionary Significance and Medical Importance: A Case of Cobra (*Naja* spp.)

**Figure 3:**
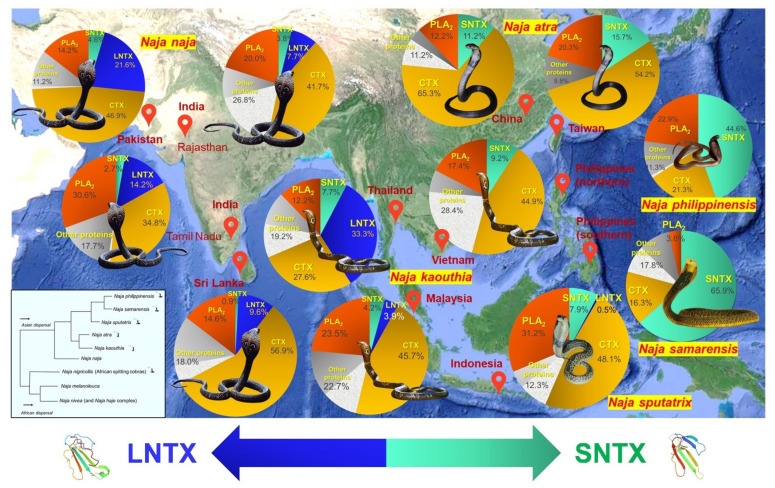
Snake venom proteomes of selected major cobra species in Asia (genus: *Naja*, subgeneus: *Naja*), investigated with venomic approaches that allow differentiation of three-finger toxin subtypes (e.g., SNTX, LNTX, CTX) and quantitation of relative protein abundances (in terms of % of total venom proteins). Genus-wide comparison and geographical mapping reveal a phenotypic venom dichotomy, characterized by the dominant expression of either SNTX (short-chain alpha-neurotoxins) or LNTX (long-chain alpha-neurotoxins) as the principal lethal toxins that mediate neuromuscular paralysis in envenoming caused by cobras. The neurotoxicity of *Naja naja* (Indian Cobra) venom is induced primarily by LNTX, while as cobras dispersed eastward, this functional role appears to be gradually taken over by the evolutionarily more derived short-chain form of alpha-neurotoxins (SNTX). In at least four occasions, there were only SNTX but no LNTX found in the venom proteomes: *Naja atra* of Taiwan, *Naja kaouthia* of Vietnam, *Naja philippinensis* and *Naja samarensis* of The Philippines. The LNTX/SNTX dichotomy has evolutionary significance and medical implications (see text). SNTX: Short-chain alpha-neurotoxin; LNTX: Long-chin alpha-neurotoxin; CTX: Cardiotoxin or cytotoxin; Other proteins include non-conventional there-finger toxins (dotted grey). Inlet shows a simplified phylogenetic tree of *Naja* cobras modified from Wallach et al. [70] and Kazemi et al. [56], illustrating the relative phylogeographical positions of Asiatic cobras (note: *N. atra* and *N. kaouthia* are considered to have partially evolved spitting behaviors). Representative structures of LNTX and SNTX were from the PDB Database (PDB entries: 1CTX and 1COE, respectively). References for proteomes: *N. naja* (Pakistan [71], Rajasthan of India [72], Tamil Nadu of India (unpublished), Sri Lanka [73]), *N. kaouthia* (Thailand, Malaysia, Vietnam) [29], *N. sputatrix* (Java of Indonesia) [53], *N. atra* (China [74], Taiwan [75]), *N. philippinensis* (northern Philippines) [51], *N. samarensis* (southern Philippines) [65].

Animal venom composition and function can vary remarkably between different species (inter-species) and even within the same (intra-species). Intra-species variation of snake venom has been widely recognized, attributed to differences in their geographical distribution, developmental stage (ontogenic influence), and sex [38,39,40,41,42]. The evolutionary drivers of variation will vary depending on the primary function(s) of the venom of individual species (or a particular population) in the context of the ecological niche that it occupies, and the extent of the variation will be partly modulated by any constraints acting on the system [43]. The ensuing functional variances can impact the venom toxicity and protein antigenicity, resulting in variability of antivenom effectiveness for the treatment of snakebite envenomation [20,44,45,46]. With venomics, it is possible to achieve high throughput profiling of different venom collections that originated from a same species, thereby shedding light on the intra-species variation. The same approach can be applied on a genus-wide scale, where the venom proteomes of congeneric species are compared for inter-species variation, especially those which form complex species [47,48,49]. A number of studies have, individually or collectively, demonstrated in a phylogeographical context the impact of venom variation on the venom toxicity and neutralizing efficacy of antivenom. An example is illustrated in Figure 3, with a reference to Asiatic cobras (genus: *Naja*; subgenus: *Naja*), which are medically important venomous snakes distributed widely throughout Asia. Genus-wide proteomics reveals the dominance of two toxin families, i.e., three-finger toxins (3FTX, constituting > 50% of total venom proteins) followed by phospholipases A_2_ (PLA_2_) in the venom proteomes of virtually all Asiatic cobras. The subtypes and relative abundances of the toxins within each family, however, vary across different species, and this has important implications on the toxic manifestation of envenoming and its treatment. In envenoming caused by cobras, both short-chain and long-chain alpha-neurotoxins (subtypes of 3FTX, abbreviated as SNTX and LNTX, respectively) are the principal toxins responsible for neurotoxicity and death, and the abundance of these toxins in a cobra’s venom is found to correlate positively with the lethal potency (gauged by the intravenous median lethal dose, *i.v.* LD_50_) of the venom. SNTX has a weaker binding affinity to human nicotinic acetylcholine receptors compared with LNTX [50], but whether or not this is translated to a lower lethality in envenoming is probably inconclusive, as both are equally potent (LD_50_ ~0.1–0.2 µg/g in rodents, intravenously), and in real envenoming where whole venom is injected, the effect will be overwhelmed by toxins that are more abundantly present [51,52]. The neutralization activity of most antivenom products against SNTX appears to be lower than LNTX, presumably due to SNTX’s limited antigenicity, but this requires further validation [53,54].

A closer look at the cobra venom proteomes and their phylogeographical relationship (Figure 3) reveals a phenotypic venom dichotomy, characterized by the dominant expression of SNTX or LNTX in venoms—as the Asiatic cobras dispersed eastward, the functional role of LNTX appears to be replaced by the evolutionarily more derived short-chain form of alpha-neurotoxins (SNTX), to the extent of virtually only SNTX are expressed in place of LNTX in the venoms of *N. atra* of Taiwan, *N. kaouthia* of Vietnam, *N. philippinensis* and *N. samarensis* of the Philippines. The Asiatic cobras (subgenus: *Naja*) are thought to be descendants of the African cobras following a single invasion (from Africa into Asia), and except for *N. naja* and *N. oxiana*, all other members of the subgenus have fully or partially evolved the spitting behavior [55,56]. Intriguingly, the African spitting cobras (subgenus: *Afronaja*) also exhibit an exclusive phenotype of biased expression toward only SNTX (in place of LNTX) in the venoms [57], in contrast to the African non-spitters of *N. haje* (subgenus: *Uraeus*) and *N. melanoleuca* (subgenus: *Boulengerina*) complexes, which produce significant amounts of LNTX [58,59]. The phenomenon indicates an alternative view of the origin of Asiatic cobras, where there are possibly two independently evolved clades in Asia, represented by the non-spitters (e.g., *N. naja*) and spitters (those with fully evolved spitting behavior, such as *N. sputatrix*, and partially evolved ones, such as *N. atra* and *N. kaouthia*), corresponding to the invasion of African non-spitting and spitting cobras, respectively.

On the other hand, all Asiatic cobra venoms contain various acidic PLA_2_, but only certain species of Asiatic cobras, e.g., *Naja sputatrix*, produce basic PLA_2_ in addition to the acidic forms. While the acidic PLA_2_ in cobra venom generally lack lethal activity, the basic PLA_2_ is lethal to mice and its toxicity possibly contribute to the cytotoxic and pain-inducing nature of the venoms of spitting cobras, perhaps in synergism with the cardiotoxins or cytotoxins [53,60,61,62]. In line with this, abundant basic PLA_2_ have also been found in the venom proteomes of African spitting cobras (subgenus: *Afronaja*) [57]. The PLA_2_, however, is not ubiquitous, as emerging evidence showed that the venoms of African non-spitting cobras (subgenus: *Uraeus*) are void of, or contain very little, secretory PLA_2_ [61,63,64]. Also, the cytotoxicity of cobra venoms has been shown to be a defensive innovation associated with hooding behavior and might have facilitated the evolution of defensive spitting in cobras [62]. Still, in some exceptional cases, such as the Philippine Cobra (*N. philippinensis*) and Samar Cobra (*N. samarensis*), their spitting behavior and the presence of cytotoxins in the venom do not fully conform to anticipated high cytotoxicity or severe tissue necrosis in envenoming [51,65,66]. This further indicates the high variability of cobra venom with regard to toxin function as well as toxin composition.

Therefore, the comparison of venom profiles across cobra species, which is made possible by venomics, unveils the importance of recognizing the inter-species variation in terms of subtypes (proteoforms) and relative abundances of the toxins. The venom toxicity and pathophysiology of envenoming can differ substantially between cobra species; hence, the treatment strategy should be tailored according to the para-specific spectrum and geographical utility of antivenom. To understand the limitation of neutralizing capacity, an antivenom product should be assessed for its efficacy and potency of neutralization against the individual lethal toxins in the venoms [67]. The production can be improved thereby based on the predominant type of toxins according to species and regionality. In this context, it has been shown that by pooling the relevant toxins from various species into a venom immunogen presenting a diverse toxin repertoire, a poly-specific, pan-regional antivenom that confers a greater neutralization spectrum against many cobras in different regions can be developed [68,69]. This approach, however, requires deep understanding of the venom composition variation of diverse snake specimens. Snake venom proteomics is thus a promising tool that can be applied, provided the methodology is well designed to capture both qualitative and quantitative details of the venom proteomes, as discussed below.

#### 3.1.2. Decomplexation vs. Non-Decomplexation Methods

The proteomic methods used in snake venomics vary from one study to another. Notwithstanding the varying methodologies, one fundamental key principle should always be observed: The work should provide identities of the proteoforms that are validated and annotated as non-redundant protein subtypes, along with their individual relative abundances that constitute the total venom proteins. Most studies, inspired by the initial venomic workflow [76], subject the venom sample to fractionation through protein separation techniques, such as chromatography and/or gel electrophoresis prior to mass spectrometry (MS) analysis (*viz.* decomplexation proteomics). In chromatography-based techniques, various types of columns can be applied (independently or sequentially, with separation based on the differences in protein ionic charges, hydrophobicity or molecular mass) but the most commonly used is a C18 reverse-phase column coupled to high performance liquid chromatography (HPLC) [24,77,78,79,80]. In a gel-based method, the venom proteins will be separated by protein differences in the isoelectric point (pI) followed by their molecular weight on a 2D SDS-PAGE (sodium dodecyl sulfate-polyacrylamide gel electrophoresis) [81,82], or simply 1D SDS-PAGE [83,84]. The mainstream and commonly used strategy for venom-decomplexation is, nevertheless, performed by subjecting the venom fractions from C_18_ reverse-phase HPLC to 1D SDS-PAGE under reducing and/or non-reducing conditions [24,85]. Often, the chromatographic separation method is preferred over the gel-based method used alone for better protein resolution and its advantage in estimating the protein abundance based on the peak area of chromatogram (area under the curve), which offers a more reliable way of quantitation than relying fully on the intensity of gel band/spot [86]. The use of HPLC also allows optimization of the elution profile through adjusting the gradient percentage of eluting buffer or solvent and the time course of chromatography. Moreover, successful identification of the protein therefrom can facilitate further studies where toxins of interest can be readily isolated chromatographically for characterization. Hence, the chromatography-based protein decomplexing method, although seemingly laborious, is rewarding: (1) It drives biodiscovery for potentially therapeutic compounds in snake venom [31,87,88]; (2) It allows the determination of principal toxin(s) in a venom, and the interpretation of the strength and limitation of antivenom products in neutralizing the venom toxicity [53,89,90,91]; (3) It provides a means of collection of key toxins from different snake venoms, thereby facilitating the reformulation of venom immunogen mixture for improved antivenom production [69].

**Figure 4:**
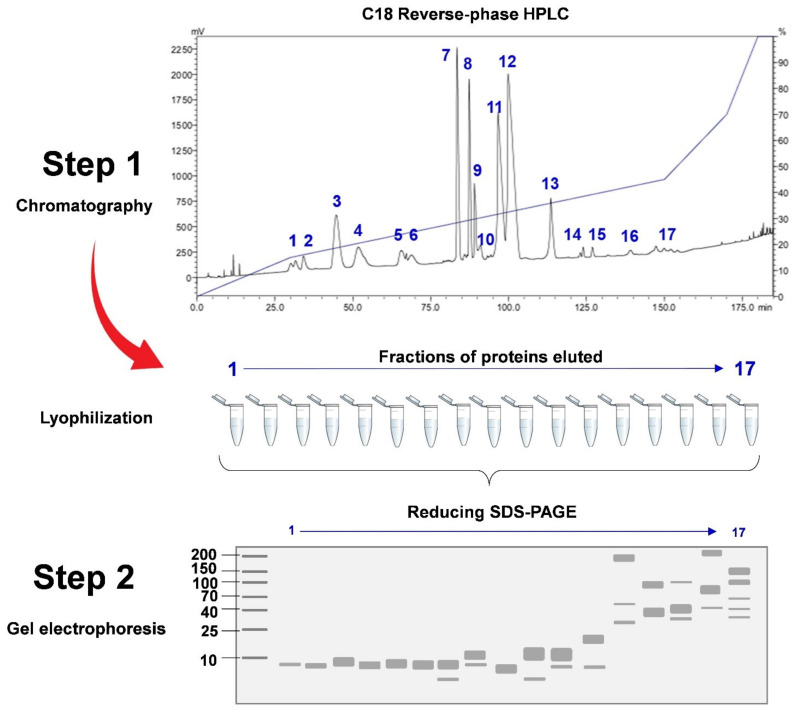
A generic venom decomplexation strategy for proteomics. In step 1, the snake venom is fractionated by reverse-phase HPLC using a C_18_ columnwith varying concentration gradients of solvent B (mobile phase) for 180 min (solvent B is acetonitrile with 1% trifluoracetic acid). The chromatographic fractions are collected manually at 215 nm (absorbance of peptide bond) and lyophilized. Proteins in the fractions are then subjected to SDS-PAGE as in step 2 (lower panel, under reducing conditions). Number 1–17 represent the numbers of chromatographic fractions collected. Protein marker is used for molecular weight calibration. The protein bands are visualized by Coomassie blue staining (Image was reproduced with reference to Tan et al. [86]).

In C_18_ reverse-phase HPLC, the venom proteins bind to the stationary phase of the C_18_ beads in the column through hydrophobic interaction. The binding by more hydrophobic proteins is generally stronger. The mobile phase is composed of an aqueous blend of water with a miscible, polar organic solvent, e.g., acetonitrile, delivered under high pressure. The flow of the solvent (mobile phase) elutes the venom proteins following a step-wise increase of acetonitrile concentration over an extended time. In a typical workflow, as the proteins are eluted, they are collected into different fractions visualized on SDS-PAGE under reducing conditions in their monomeric form (Figure 4) [86]. On the other hand, in the non-decomplexation study, the venom is not subjected to any biochemical fractionation right from the beginning (Figure 2). Apparently, in contrast to the more laborious and elaborated decomplexation technique, the non-decomplexation approach is useful for venom samples that are available in only a minute amount, or the supply is limited, in particular from some exotic species [49,83,92,93]. The non-decomplexation method also allows quick profiling of the venom when time, resources, and budget are limited (see Table 1 for comparison). The venom proteins, either decomplexed (separated as chromatographic fractions or gel spots/bands) or retained whole (non-decomplexed), are then analyzed by mass spectrometry (MS) for peptide sequencing, adopting the conventional bottom-up or the emerging top-down sequencing techniques (discussed below). The MS data is then processed by bioinformatics and database searching for protein identification and quantitation.

#### 3.1.3. Bottom-Up, Middle-Down (Middle-Up), and Top-Down Sequencing Strategies

**Figure 5:**
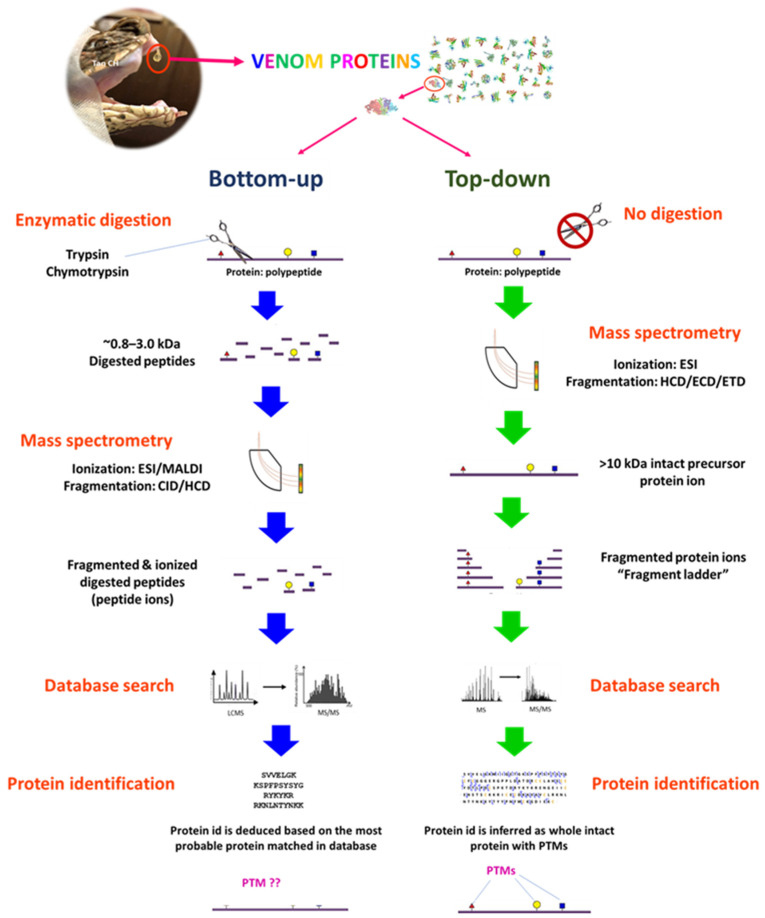
Bottom-up and top-down proteomics in snake venomics. The stark difference between the two approaches is whether or not proteins in the venom are subjected to proteolytic digestion prior to mass spectrometry (MS) analysis. In bottom-up proteomics, the proteins are digested enzymatically into short-length peptides that are then ionized in MS, fragmented, and the peptide masses are deduced. Their empirical peptide masses act like “fingerprints” that are subsequently correlated with known proteins in databases using search engines, such as Mascot or Sequest. Protein is identified indirectly based on sequences of the tryptic peptides that are assigned to reconstruct, though incomplete, a protein. In top-down proteomics, the intact proteins are ionized whole and then fragmented by MS, and the masses of the ionized proteins and fragments are analyzed to inform on the full sequence of the proteins along with important post-translational modifications (PTM).

The use of mass spectrometry (MS) is integral in any venomic work as it is the high-throughput mean by which the toxins can be identified confidently as non-redundant proteins, and in many instances, it facilitates the quantitation of protein abundance [34,63,71,95]. MS is needed for the ionization of venom protein (intact) or digested peptide of the venom, separation, and detection of the protein/peptide ions by their mass-to-charge ratio (*m*/*z*) in a gas phase. The electrospray ionization (ESI) and matrix assisted laser desorption/ionization (MALDI) are currently the most employed ionizing techniques used in MS for venomics. ESI requires liquid phase samples, and thus is ideal for online coupling with liquid phase separations, such as liquid chromatography (LC) and capillary electrophoresis (CE). MALDI and ESI techniques are complementary and can thus be used in parallel in order to obtain a venom profile that is as exhaustive as possible [29,76,100]. As the ionization process in MS is a critical step in amino acid sequencing for the protein/peptide, one should ask whether the MS in use is capable of the following tasks: (a) Can it sequence the protein in its truncated forms or fragmented peptides, which are to be joined and re-assembled then? (b) Can it sequence the whole protein by its intact form without prior modification that alters its complete protein structure? Both sequencing strategies are aptly called the bottom-up and top-down proteomics, respectively, and have been applied in venomic studies. The basics of the two MS-sequencing strategies are illustrated in Figure 5 and discussed further in the context of snake venomics.

Conventional methods of protein truncation subject the proteins to enzymatic proteolysis (e.g., trypsin, in preparation for peptide ionization and detection by MS) or chemical degradation (e.g., cyanogen bromide, in preparation for Edman’s degradation which is not a practical choice for high-throughput venomic works). Protein identification through sequencing its truncated form (peptide or polypeptide) obtained by proteolysis is referred to as a “bottom-up” approach. This approach is mature, well established, and widely adopted in almost all MS-based proteomic fields, including venomics. Three biochemical modification steps are conducted on the protein mixture (venom) prior to LCMS and MS/MS analysis: protein reduction, alkylation, and enzymatic digestion [86]. The latter is usually achieved with trypsin, although other proteases, such as, chymotrypsin can also be used instead or in parallel to generate peptides within ideal mass range and charge state for ionization, and in instances where certain proteins resist trypsin digestion (such as the proline-rich waglerin peptide in *Tropidolaemus wagleri* venom) [38]. Complete enzymatic digestion of the protein will yield numerous peptides of length between 7–20 amino acids (0.8–2 kDa), cleaved at specific amino acid sites depending on the protease used. A nano-scale chromatographic method is then applied to separate these peptides in complex mixture prior to MS, an integral technique commonly referred to as liquid chromatography-mass spectrometry (LCMS) [101].

Naturally, depending on the complexity of a venom sample and the resolution power of the pre-MS chromatographic method employed, not all peptide fragments would be detected during MS analysis [102]. The number of peptide spectra generated compromises the sensitivity of bottom-up MS, and it is estimated that as much as 75% of spectra could remain unidentified due to several factors, such as low signal-to-noise events, incomplete databases, and unexpected post-translational modifications (PTMs) [103]. Therefore, it is virtually impossible to recover full sequence coverage of a toxin protein through bottom-up proteomics, more so when the database of a specific venomous species is unavailable due to paucity in its de novo genome and transcriptome sequences. This will in turn limit the detection of proteoforms (non-redundant subtypes that show protein diversity within a venom) and important PTMs that give rise to different isoforms. Protein identification following MS analysis relies on a database search that matches the in-silico peptide sequences by homology or similarity shared by other proteins from various other species, akin to assembling a puzzle. High-efficiency automated bioinformatic protocols are available to accomplish the matching, and the results are then ranked by scores that compare empirical spectra to theory. Nevertheless, in the absence of a complete sequence of protein for a specific species in query, the protein identity informed by such algorithms must be carefully scrutinized with additional maneuvers, which include eyeballing (literally) all peptide sequences assigned per matched proteoform (toxin), applying stringent filtering criteria, such as having ≥2 distinct peptides for each protein matched, and accepting only annotations of reasonable, phylogenetically related species/genus/family [47,94]. These additional steps are advisable in order to minimize invalid and redundant protein identification in bottom-up proteomics for species whose genome sequences are yet to be available.

In contrast, the top-down technique involves the sequencing of intact protein without resorting to truncation (essentially, enzymatic digestion), and Edman’s N-terminal sequencing aptly fits into this. In the current practice where MS is used, top-down proteomics has the advantages of direct detection of the native mass of a protein, and possibly retrieval of its full amino acid sequence along with sequence variation and PTMs in isoforms [104]. However, the versatility of this approach is restricted by its technical difficulty, requiring high throughput technology and advanced equipment or programs, which are less commonly used or simply not compatible with most of the existing MS. The successful application of the top-down strategy is critically reliant on MS fragmentation methodologies, which must be sufficiently efficient and optimally fine-tuned in order to reproducibly fragmentize low and high molecular weight peptides [102]. This method is about 100-fold less sensitive than the bottom-up technique and has a lower efficiency and throughput. The technique, in brief, involves gas phase ionization of intact protein(s) with ESI, and the protein ions are subsequently fragmented by collision-induced dissociation, or the more delicate electron-capture dissociation and electron-transfer dissociation methods in the mass spectrometer. The ions of both intact and fragmented proteins are detected based on their masses, and the sequences are deduced from database searches. By “bridging” multiple sufficiently long enough fragments, it aims to uncover the protein’s primary structure along with its PTMs. However, gas-phase fragmentation of intact protein ions for high molecular weight proteins larger than 50–70 kDa is difficult, and a high-end instrument is needed to resolve the differences between large molecules of similar sizes [103]. The instrumentation for dissociation and fragmentation required for use in top-down proteomics, e.g., ECD (electron capture dissociation) and ETD (electron transfer dissociation) integrated with tandem mass spectrometry, are costly and are technically low-efficiency processes requiring long ion accumulation, activation, and detection times.

Not surprisingly, the top-down sequencing strategy is uncommonly applied in venomics despite its attractiveness in terms of potential access to all proteoforms and their full sequences, as well as PTMs. The first top-down proteomics was reported for Indonesian King Cobra (*Ophiophagus hannah*) venom [79]. The study showed a venom composition that varied substantially from other King Cobra venom proteomes reported previously using the bottom-up approach (specimens from Malaysia, China, Thailand, and Indonesia) [97,105], with the high molecular weight proteins being of note. L-amino acid oxidases (LAAO), which are usually present abundantly in King Cobra venom, was identified at an exceptionally low abundance level (one LAAO form, 0.5% of total venom proteins) in the top-down proteomic study [79]. Nonetheless, from the venoms of two African mamba species (*Dendroaspis polylepis* and *Dendroaspis angusticeps*), the top-down method was able to unravel the extreme diversity of toxins where more than 200 polypeptides were identified, including previously undetected protein species, isoforms, and proteoforms [106]. This was followed up by another study that also characterized the proteome of King Cobra venom, applying top-down proteomics under different experimental conditions [107]. The later study showed that different top-down methods resulted in highly variable proteoform detection from the same venom sample—in the extreme case, the benchmarking LAAO was not be detected in the whole venom proteome [107]. The study suggests that top-down proteomics may have limitations for analyzing intact proteins that are larger than ~50 kDa, e.g., LAAO (and perhaps also other high molecular weight enzymes), a condition that has also been recognized by others in non-venom samples [103]. The study showed that the native condition is probably the most optimum experimental condition for top-down proteomics, as it could overcome the limitations in studying the glycoforms of large toxins, which the bottom-up approach does not [107]. Table 2 summarizes and compares the applications of the bottom-up and top-down approaches applicable for use in snake venomic studies.

Apparently, detecting more unique peptides of greater lengths would help to retrieve the full sequence of a venom protein, and therefore allow a closer look into the diversity and PTMs of toxin proteoforms therein. Yet, beyond a certain length of a long peptide, or as in the case of intact high molecular weight protein, such as LAAO, it is rather clear that the resolution and detection of large protein/fragment ions by MS would be greatly compromised. Therefore, between the bottom-up and top-down approaches, there is possibly an alternative strategy that strikes a balance—the middle-down approach is thus proposed [102,108]. One may argue though that it should be called the middle-up approach; after all, it shares more characteristics with the bottom-up technique. Instead of an intact protein, it creates truncated peptides by proteolysis, while keeping the length of the digested peptides greater (20–100 amino acid residues, 2.5–10 kDa) than those from a conventional bottom-up approach (~7–20 amino acid residues, 0.8–2 kDa) [108]. The number of fragmented peptides in a sample produced by a middle-down approach would be relatively smaller, and thus less complex compared with those from the bottom-up approach. This theoretically would enhance the detection of more distinct peptides without being limited by an overly long peptide sequence, as in the top-down method. The middle-down approach, nevertheless, requires restricted enzymatic proteolysis with special enzymes, such as the outer membrane protease T (OmpT) [109]. This approach has not been reported in snake venom proteomics, and investigation comparing the proteome outcomes of the three approaches (top-down, middle-down and bottom-up) would be meaningful in future venomic research.

### 3.2. Genomics and Venom-Gland Transcriptomics of Snake

Although the venom proteomes of most major snake species have been widely characterized (with varying depths and details), the knowledgebase created is largely confined to composition profiling (cataloguing), and even so, many debatable issues remain unresolved with regard to the identification and quantitation of toxin proteoform [34,110]. The ambiguity of toxin identification is partly due to the inadequate understanding of the genetic bases of snake venom, which is a pre-requisite to elucidate the evolution of venom, diversity of toxins, and regulation of toxin production. To remedy this, genomics and transcriptomics have been increasingly adapted and incorporated in venomic studies [111,112,113]. Also, the availability of species-specific datasets built from de novo genomics or transcriptomics will correct for the absence of unique peptide sequences in public databases, hence improving the accuracy of toxin identification in proteomics for a more comprehensive profiling of snake venom diversity.

Unlike venom proteomics that deal with the secreted venom, genomic and transcriptomic studies utilize body tissues including the venom gland, from which RNA and/or DNA are extracted. In this primary process, the tissue-harvesting skill is critical, and fresh tissue samples are generally preferred to ensure high integrity of the DNA or RNA [114,115]. The process, in brief, will be followed by cDNA library construction, sequencing, functional annotation, and gene expression study (transcriptomics) (Figure 2). In all, the nucleotide sequencing technique remains central. It should be credited that the explosion of genomic and transcriptomic data over the past decade is accelerated by the advancement in sequencing technology and the expansion of bioinformatic databases. Earlier, the very first generation of DNA sequencing was done with the Sanger technique, a chain termination method based on the selective incorporation of chain-terminating dideoxynucleotides by DNA polymerase during in vitro DNA replication [116]. It is a well validated but expensive, laborious approach, and has remained in limited use for small-scale projects, and for validation of alternative sequencing techniques. The next-generation sequencing technique allows mass parallelization of sequencing reactions, increasing the amount of DNA/RNA that can be sequenced in any one run (i.e., high throughput characteristics). This began with the second-generation sequencing technique, e.g., pyrosequencing, and was followed by a number of parallel sequencing platforms which drastically decreased cost, increasing flow rate and attractiveness of DNA/RNA sequencing. For years, the Illumina sequencing platform has been the most widely used, almost to the point of monopoly [117]. The “short-read” sequencing technologies, such as Illumina platforms, have lower error rates and can provide highly accurate genotyping in non-repetitive regions but do not allow contiguous de novo assemblies. This restricts the ability to reconstruct repetitive sequences and to detect complex structural variation [118]. Currently, the third-generation of NGS technologies are available for whole genome sequencing (WGS). The three commercially available platforms most commonly used are the Pacific Biosciences (PacBio) Single Molecule Real Time (SMRT) sequencing, Illumina Tru-seq Synthetic Long-Read technology, and the Oxford Nanopore Technologies sequencing platform (MinION), which allow direct sequencing of single DNA molecules to produce substantially longer reads than second generation sequencing [119]. The third generation sequencing alone, however, is prone to having high error rates; hence, complementary short-read data (such as that sequenced by Illumina technology) is often incorporated for high-quality de novo genome assembly [118,120].

In the venomic field, the honeybee, *Apis mellifera*, marks the first venomous animal whose whole genome was successfully sequenced, as reported by the Honey Bee Genome Sequencing Consortium (2006), though the study focused on its ecology and biology context instead of the venomous system [121]. The genomes of snake were available much later in 2013−2014, with the first drafts to be published from the Boa constrictor (*Boa constrictor*) [122], Burmese python (*Python molurus bivittatus*) [123] (both python and boa are non-venomous snakes), the venomous King Cobra (*Ophiophagus hannah*) [124] and colubrid Corn Snake (*Pantherophis guttatus*) [125]. Over the years more genomes of snakes were reported, accompanied with increasingly sophisticated sequencing technologies and higher coverage assembly. These include colubrid such as the Garter Snake [126], and venomous species such as the Five-pacer Viper [127], Okinawa Habu [128], Indian Cobra [120], Tiger Rattlesnake [129] and sea snakes (*Hydrophis cyanocinctus* and *Hydrophis curtus*) [130] published more recently. A search for snake genome assemblies (infraorder: Serpentes, Taxonomy ID: 8570) deposited in the NCBI database recalled 39 projects (as of March 2022), of which 24 belonged to front-fanged venomous snake species, 13 were of mildly venomous or non-front-fanged snakes (with distinct and repeated species), one is a non-venomous constrictor snake (python), and another is a blind snake (Table 3). Obviously, the number of venomous snake species with full genome sequenced to date is small but the available findings showed that snakes, regardless of body size and venom-producing character, share a relatively small genome size (~1.3–1.8 Gb) that is closer to those of other sauropsids, e.g., the anole lizard (1.70 Gb) [131] and avians (birds) (1.05–1.26 Gb) [132,133], but relatively smaller (half the size) comparing with the human genome (3.54 Gb).

The next in genomic or transcriptomic pipeline is functional annotation, i.e., attaching relevant biological information to the sequences, and predicting the gene’s or protein’s identify. The advent of high throughput gene sequencing greatly facilitates the prediction of all translated genes (exome) by automated programs (e.g., ab initio gene prediction tools) [134], and homology searches using reference sequences [135]. The sequencing reads are aligned and mapped to reference genomes by mapping programs, such as the Burrows-Wheeler Aligner (BWA) [136] and Bowtie [137]. In cases where genome sequences were unavailable, as with the majority of snake species, reads can be translated into protein coding sequences and subjected to homology searches against publicly available databases, such as COG [138] and Pfam [139], applying database search tools such as the widely used Basic Local Alignment Search Tool (more commonly known as BLAST). BLAST is an online computer algorithm available free at the National Center for Biotechnology Information (NCBI) website, and is widely used for comparing and calculating similarity of primary biological sequence information from venom protein (amino acids) and snake DNA/RNA (nucleotides) to infer the most probable putative toxins.

Information from de novo venom-gland transcriptomics can be useful to some extent for gene prediction of translated proteins (also, microRNAs (mRNAs) and other non-coding genes), since duplication of toxin-encoding genes is common in closely related species. Transcriptomics also has an advantage in determining the differential expression of genes for both toxin and non-toxin copies [21,114,115,140,141,142,143]. With reasonable investment of time and cost, it is by far the most practical strategy for comparative venom gene profiling across multiple specimens. However, the genetic content derived from venom-gland transcriptome is obviously smaller than full-scale whole genome, hence its use is limited for elucidating the origin and mechanism of venom evolution in snakes. High-quality genome assembly and comprehensive annotations of venom protein genes, as well as highly similar non-venom paralogs from not only the venom-gland tissue but also different parts of the body are warranted in future venomics to address deeper questions surrounding venom evolution. For instance, one fundamental and debatable questions is: *what are the “real toxin” genes of venom?* Full de novo sequences allow gene analysis for positive or negative selection (as inferred by the *d*_N_/*d*_S_ ratio) to identify those undergoing accelerated evolution in keeping with the function of venom toxin [144]. The snake venom phospholipase A_2_ (svPLA_2_) represents a classical example: Nakashima et al. demonstrated earlier that accelerated evolution occurs in the protein-coding regions (exons) of pit viper svPLA_2_ genes, consistent with the multiple forms of the enzyme with diverse biological activities in the snake venoms [145,146]. Over the years, abundant evidence continues to show that multiple genes are under selection in snakes, or in clades within the snakes, including toxin genes in venomous snakes [120,124] and developmental genes possibly connected to development of the serpentiform body plan in non-venomous species [123]. It is commonly believed that the toxin genes were co-opted from body’s physiological proteins [17,147]; however, the preexisting gene elements (from which the specific toxin gene arose), and the mechanism by which the ancestral genes transformed into toxin genes with unique protein domains, remain to be studied in different snake species.

As mentioned, proteomics has extensively demonstrated variation of snake venom composition between and within species. Some of the factors associated with the variation, in particular at the intra-species level, include geographical origin and developmental stage of the snake [1,20]. However, snake venom variability is itself inherently variable, and does not necessarily conform to reported variations. Despite knowing the associated “factors”, the mechanism of venom variation and its consequent impact on function and toxicity have not been well elaborated in most instances, ostensibly due to the paucity of genome sequences of most snake species. How did the snake venom proteins diverge, structurally and functionally between and within the various species? Gene duplication followed by neo-functionalization is a generally accepted hypothesis [148], while there are also views that point to transcriptional and post-transcriptional regulations [149], in addition to gene loss or pseudogenization (degeneration of functional genes, which can be identified in the genome sequence on the basis of sequence homology or synteny across species) [150,151], among various mechanisms proposed.

Therefore, high-quality genomes of venomous snakes, when combined with transcriptomics and proteomics will contribute to significant advancement in venomics. Species-specific toxin genes along with the expressed proteoforms can be established, and the data would be useful for a variety of applications, including probe design and in situ hybridization [152], and identification of toxin gene regulatory regions at genomic scale [129]. In studies where the snake genome was assembled at chromosome level, it is possible to better understand structural variation or rearrangement of genes (e.g., inversions, insertions, deletions and tandem duplications), and to determine the loci of duplicated genes (clustered or scattered) [153,154] as well as transposable elements and other repetitive sequences [148] that a venomous snake acquired during evolution. The genomic data can also be used in phylogeny reconstruction (as in phylogenomics) while bearing in mind that the accelerated evolution of toxin nucleotide sequences might obscure the ancestral sequences or the reconstruction could be further compounded by extensive changes in genomic content following gene loss and gene duplications [151,155]. From the perspective of medicine, the identification of toxin genes and the resolution of their sequences through genomics and transcriptomics will theoretically provide valuable information for the development of next-generation antivenom. By uncovering the toxin epitopes based on genome sequences, recombinant and synthetic monoclonal antivenom can be produced in vitro against a certain species from a specified locale [120,156]. Alternatively, the targeted toxins of various species from multiple locales can be determined and consolidated as a new immunogen formulation for the development of a pan-region, poly-specific antivenom [68,69]. Furthermore, information from genomic analysis will encourage the exploration of venom protein structures on a genome scale (structural genomics), for a deeper understanding of the structure-activity relationship of toxins and their physiological targets, as well as how generic inhibitors can be devised as new antidotes to treat snakebite envenoming [157,158].

## 4. Challenges and Recommendations

### 4.1. Sampling

Venomics seeks to profile the global composition of a venom, specifically, the secreted toxins (proteins) which can be determined directly by sequencing their amino acids (proteomics), or indirectly by sequencing the mRNA/cDNA (transcriptomics) responsible for the expression of the proteins, or through full species genomics. In this regard, the sampling is crucial to warrant the validity, reliability, representativeness and reproducibility of the studies. However, the collection and processing of venomic samples are challenging (as discussed below), and the difficulties often constitute the major limitations or become causes of controversy in some studies. Consensus among researchers in the field on issues pertaining to the collection and processing of venomic samples will help reduce the problem but realistically speaking, it is easier said than done when one puts things into practice. Scientists would often have to resort to and make the best out of what is available without much compromising the quality of the research.

#### 4.1.1. Availability and Authenticity of the Sample

Virtually all venomous animals are wildlife. The sourcing of sample, be it the venom (for proteomics) or body tissue (for transcriptomics and genomics), is subject to the obtainability of at least one live specimen that is in good health conditions, and the success of manually collecting the sample by trained personnel who extracts the venom or dissect the tissue from the animal. Some specimens are kept in captivity or farmed and are thus commercially available, while most other species remain far from reach in the wild. The specimens, be it farmed or wild, must be correctly identified down to the species (or even subspecies) level according to the latest taxonomy. Depending on the number of specimen available for a species, the size and nature of the sample varies from one study to another. Often, venoms are extracted at least once from each specimen and subsequently pooled for two justifications: (1) To ensure a substantial amount of venom sample is available for study; (2) To “average out” the variability between individual specimens for a result that is even and representative of the species. Certainly, this pooling approach is not without weakness as it basically “destroys” any inter-individual variation which could be ecologically and medically important. On the other hand, transcriptomic and genomic studies typically involve the use of one single specimen which provides the tissue sample. Commonly, the proteome of venom from the individual specimen is also characterized for correlation and interpretation of toxin gene expression.

It is therefore of great importance to have collaboration between laboratory scientists (e.g., biochemists, pharmacologists) and field researchers (e.g., herpetologists, marine scientists) to ensure the accessibility of authenticated specimens in venomic sampling. Considering the difficulty in obtaining sufficient specimens, a small sample size is often justified but the number should be explicitly stated in the work for future references. Comparing the venom proteomes of individual specimens may provide some insights into inter-individual variation but the work can be rather laborious and costly yet not necessarily providing a representative profile of all specimens tested. This should be considered only when there is a clear indication of potentially significant variation between individuals secondary to influences such as geographical distribution, ontogenic shift, seasonal effect, sexual dimorphism, captivity (vs. wild) and so on.

#### 4.1.2. Batching, Referencing, Storage and Quality Control of Samples

The venoms or tissues collected should ideally be kept separate per individual specimen and made distinguishable from batches of pooled samples. In studies involving the use of body tissue, the venom of the individual should be obtained, and a set of reference sample constituting the venom and tissue sample is kept. Often, the collection of certain vital tissue/organ will inevitably result in euthanasia of the animal, so whenever possible, the remains of the animal should be kept as a voucher specimen for record verification.

Systematic archiving of sample for traceability and standard operating procedures for sample handling, transfer and long-term storage are important aspects in venomic studies. Stringent quality control measures should be implemented to ensure the sample (venom or tissue) tested are in the most original form with the least possible degradation and contamination. For venom sampling, the common practice currently in the field is to minimize possible protein degradation following venom extraction by immediately keeping the venom collected at low temperatures (e.g., by submerging in ice, snap-freezing in liquid nitrogen or dry ice) and transferring the sample at the soonest for lyophilization. Alternatively, venom samples may be desiccated with a desiccant like silica gel and calcium chloride where resources for freezing and lyophilization are unavailable. The lyophilized or dried venom will be reconstituted in appropriate solutions then for use. Unused reconstituted venom stock may be refrozen for re-use but repeated cycles of freezing-and-thawing can potentially destroy some protein components and thus reduce the biological activities of the venom.

Tissue samples are more delicate and the genetic materials, in particular RNA are readily degradable. The tissue should be obtained from a live specimen (under proper anaesthesia or immediately after euthanasia) whenever possible, and the tissue needs to be preserved with an agent compatible for downstream analysis. For genomic samples, the tissue can be readily kept in undenatured absolute ethanol whereas for transcriptomic samples, besides ethanol, a stabilization and storage solution is available for use (e.g., RNAlater solution). To maximize the preservation of the genetic materials, the tissue sample needs to be excised to increase the surface area for optimal permeation of the stabilizing solution. The permeation should be allowed to take place at least overnight, and the tissue samples can then be kept (with or without the solution) below zero degree Celsius for long-term storage. Cycles of repeated freezing-and-thawing of tissue sample should be minimized.

#### 4.1.3. Ethics Approval and Permit Requirement

Ethics regulation is applied to the use of most vertebrate laboratory animals for research, and in this respect sampling of venom or tissue from venomous animals has been subject to ethical approval in some institutions. Accordingly, standard protocols need to be put in place to safeguard the welfare of the animals and the safety of users. As most venomous animals are wildlife, permits for use may need to be obtained from relevant authorities too in some cases. Transfer of sample across borders is often subject to special inspection and clearance by the immigration department. These are measures increasingly adopted by the scientific community for safe science and better research integrity, and the practice is commendable. Relaxation of certain regulations, however, should be considered on case-to-case basis in some situations for limited resources and technical supports as well as the pressing need of the research in specific areas. Often, this refers to strict regulations of wildlife use where sampling is prohibited to begin with. In some countries, transfer and sharing of wildlife-derived research materials across borders are not even allowed, and this greatly impacts research collaboration internationally. This inevitably leads to delayed knowledge transfer and pre-empts scientific discoveries, impeding the progress of various research efforts and resolutions such as those aiming to advance medical care for snakebite in neglected populations, and to improve ecological conservation of venomous fauna. The solution is perhaps to “soften” the inflexible rule by bringing awareness to the government and public including NGOs regarding the significance and urgency of the work, so that international research collaboration can be duly recognized and facilitated by the authority. In this effort, the WHO and relevant global organizations including funding bodies play an instrumental role—experts from various regions, including those of less developed world should be fairly recognized and included as representatives in snakebite-related working groups, advisory panels or taskforces initiated by these international organizations; for instance, the WHO Working Group on Snakebite Envenoming, and the Global Snakebite Initiative. Due recognition of expertise in the field will help strengthen global collaboration on promoting sampling, material and technology transfer, and data sharing across borders.

### 4.2. Protein Quantitation

Venoms are complex adaptive traits of animals and therefore variable among organisms under distinct evolutionary pressures. The natural philosophy of the phenomena (i.e., the observation and its qualitative reasoning) form the foundation for most venomic studies, which further expounded the temporal and spatial patterns of venom variation through empirico-mathematical investigation. Venomics has since moved beyond gene and protein identification, that in any such study it is expected to also unravel the complexity of proteoforms along with the quantitative measurements of their quantities, or expression levels. High dynamic resolution has been well established for genome-wide gene expression either with RNA microarrays [159] or next-generation sequencing [160,161], but in the case of venom proteomics the quantitative analysis is much trickier and more challenging. Proteomics essentially relies on the use of mass spectrometry, which is not inherently quantitative due to differences in the ionization efficiency, detection sensitivity and incomplete databases that compromises gene/protein identification. Various methods have been innovated and adapted to overcome the analytical limitations (as reviewed in [33]) but needless to say, there is no “one-size-fits-all” method that can claim the comprehensiveness of all proteins detected and quantified. Consequently, a variety of quantitative analyses were applied in venomics and reported. There is apparently no clear consensus, the neglect of which has, the author supposes, been at the root of much of the controversial discussion and conflicting views among toxinologists as to how proteins should be quantified in a venom proteome. This review does not intend to criticize the different methods adopted by individual research groups, but instead attempts to address the acceptability (or rejectability) and potential impact of the application of different quantitation methods in venomics.

#### 4.2.1. Quantity of Protein: How Much, or How Many?

**Figure 6:**
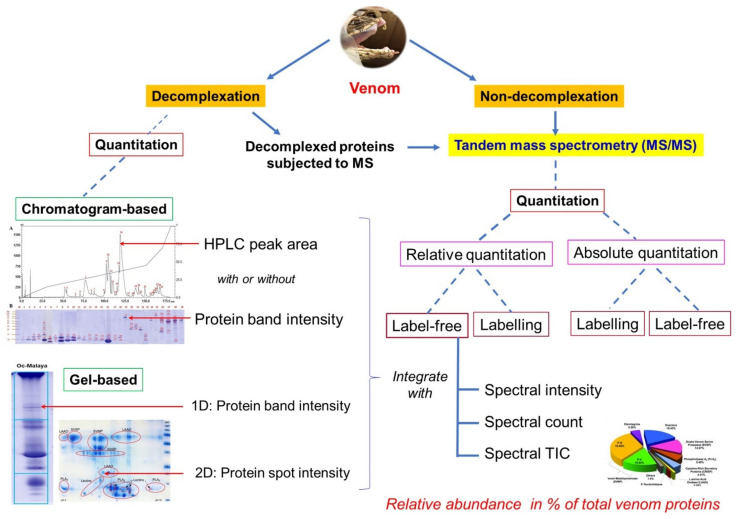
Protein quantitation in snake venom proteomics. Proteomics is studied either with or without protein decomplexation (by HPLC and/or gel electrophoresis) prior to mass spectrometry analysis for protein identification and quantitation. The label-free, relative quantitation approach is the most commonly used. The relative protein abundance of venom composition is interpreted based on individual protein’s spectral intensity, spectral count, or spectral total ion current (TIC) (integrated with HPLC peak area and/or gel intensity, quantitative parameters from venom decomplexation if relevant). Images of HPLC, gels and pie chart for illustration were adapted from previous studies [73,93,162].

In a typical venomic workflow, venom components identified (following mass spectrometry analysis) are catalogued into a list of non-redundant proteins. Naturally, the quantity of each protein that constitutes the venom proteome needs to be resolved. In most studies, the term *relative protein abundance*, expressed in percentage of total venom proteins is used to denote the proportion of a protein in the venom proteome. The most commonly used method is one that builds on information derived from the protein decomplexation steps (e.g., chromatogram peak area, gel band/spot intensity), and mass spectrometry analysis (Figure 6). In snake venom proteomics, the relative quantitation analysis with label-free technique is the most common. This is a peptide-centric method, which assumes that the more abundantly a proteoform is present in the venom (in terms of mass), the higher its peptides’ spectral intensity and/or spectral count as analyzed by label-free mass spectrometry. Where there is prior decomplexation of the venom, the proportion of the spectral intensity or spectral count will be further adjusted according to the chromatogram’s peak are (area under the curve), and/or the intensity of protein band or spot under gel electrophoresis (Figure 6). By integrating chromatography peak area and/or gel intensity in estimating the relative abundance of protein, bias from peptide-centric quantitation by mass spectrometry may be reduced.

In contrast, another method of quantifying the proteome is by calculating the *ratio* of a protein, whereby the total number of proteins identified in the venom is set as the denominator. This quantifying method is incongruent with the concept of protein abundance determination, as it basically represents the number or occurring frequency of a protein species found against the total number of venom proteins (i.e., *how many?*) without an indication about its proportion by mass that contributes to the bulk of all venom proteins (i.e., *how much?*). Calculation via either method can result in remarkably different quantitative profiles of the venom proteome. Using the data recently published for the venom proteome of *Trimeresurus puniceus* (Indonesian Ash’s Pit Viper) [47], Table 4 shows the differences between two sets of quantitative data derived from the different calculating methods, as mentioned above.

Note that in Method 1, the relative abundance of each non-redundant protein was estimated based on the spectral intensity of its unique peptides derived from MS analysis, integrated with the peak area of chromatographic fraction in which the protein was eluted. Method 2, however, is independent of the peptide’s spectral intensity and spectral count. Instead, each protein was given a fixed ratio of 1:59 (the total number of all non-redundant proteins identified in the study was 59), or simply 1.69% as its “frequency of occurrence” among all 59 proteins identified. This method of “quantitation” is misleading, and it does not provide much meaningful information with regard to the relative abundance of a toxin in the venom. Consequently, the cumulative percentage of proteins belonging to the same family cannot correctly reflect the true composition of the venom.

The generic formulae for Method 1 and Method 2 are, respectively, shown as follows:

Method 1 (considering the use of HPLC for venom protein decomplexation):
  (1) $$Relative\;abundance\;of\;a\;protein\;in\;\;an\;HPLC\;fraction\;\left(\%\right)\;=\frac{Mean\;spectral\;intensity\;of\;protein\;in\;a\;fraction}{Total\;mean\;spectral\;intensity\;of\;a\;fraction}\times 100\%$$
  (2) $$\begin{array}{l} Relative\;abundance\;of\;a\;protein\;\left(\%\right)\\ =\frac{\%\;AUC\;of\;a\;fraction}{Relative\;abundance\;of\;a\;protein\;in\;a\;fraction\;\left(\%\right)}\times 100\% \end{array}$$
Method 2 (regardless of decomplexation or non-decomplexation):
  (3) $$\begin{array}{l} Relative\;abundance\;of\;a\;protein\;\left(\%\right)\\ =\frac{Number\;of\;the\;protein}{Total\;number\;of\;all\;proteins}\times 100\% \end{array}$$

#### 4.2.2. Quantifying a Protein without Model Organism

In label-free quantitation by mass spectrometry, the relative spectral peak intensity or spectral count is used as a surrogate of the protein abundance with two assumptions: (1) The probability of data-dependent precursor ion selection is higher for abundant precursor ions; (2) the number of peptide identifications are normalized to account for the fact that larger proteins tend to contribute more peptide/spectra [163]. The label-free measurements were long thought to be reliable methods for quantifying protein abundance changes, particularly in shotgun proteomic analyses [164]. The methods have also been adapted for protein abundance estimation in snake venomics, credited to the availability of mass spectrometry with improved resolution for better peptide detection, and the expanding databases in recent years that complement protein identification. The approach is more efficient than labelling proteomics, and is increasingly gaining popularity as advancements in genomics and transcriptomics begin to overcome the limitation of proteomics in studying non-model organisms [165]. Nevertheless, the approach has sometimes come under criticisms as label-free quantification methods arguably work only for model organisms whose genomic or transcriptomic databases are available [34]. According to the disagreeing view, protein identification is a limiting factor in label-free quantitation, since, in the absence of genome and transcriptome of the species serving as the model organism, the protein identification will not be comprehensive to allow for label-free quantitation. It is, however, debatable as the present databases are deemed by many to be sufficiently mature and comprehensive for protein identification purposes, provided stringent filtering criteria are applied to discern distinctive and non-redundant proteoforms. This is believed to hold true for at least most of the major clades of venomous snakes, as their venom proteins share highly conserved structures (sequences) to allow for reliable identification. In fact, even though a “model organism” database is available, most of the time it is still impossible to identify *all* proteins in the venom *comprehensively*, as the protein identification is critically dependent on the depth of the single database used (that represents the much-insisted model organism), and any significant genetic variation between the sequenced organism (typically a single snake) and those which contribute to the venom pool. In author’s experience, when a search is restricted to only the database of a model organism (i.e., the species), the yield of peptides detected and the number of proteins identified would be smaller than results obtained from a search protocol that integrates databases of the model organism and those from public domains that contain homologous sequence information of other related species. It appears that the databases are complementary to each other, and their use should not be mutually exclusive. The insistence on the model organism’s database is reasonable, but without which, it should not be the reason to reject venomic findings that were analyzed using publicly available databases. In fact, the beauty of venomics, as it was initiated more than a decade before, is that venom proteomics was made possible (virtually for any venomous snake species) by the evolving technologies—the advancing MS techniques, sophisticated bioinformatic tools, and the ever-expanding biological databases. Snake venom proteomics was actually accomplished even before the genome or transcriptome of any particular venomous snake species was available.

#### 4.2.3. Reconciling the Divergence

At present, the wealth of public databases, bioinformatic tools, and protein search algorithms have greatly facilitated the profiling of protein diversity in snake venom. Viewpoints regarding protein quantitation in venomics remain divergent and often conflicting among toxinologists. The issue cannot be resolved without reaching a consensus on the terminologies used, and without knowing the pros and cons of analytical methods applied. The following suggestions are made for reconciling the divergence:

(i) Each *protein* regarded as a distinct form (proteoform) should be identified based on the presence of unique peptide(s) not otherwise shared with other known proteins or isoforms. While homologous sequences are assigned by search engines to different proteins, these sequences should be inspected meticulously to see if they are indeed representatives of distinct proteoforms, or simply mergeable into one whose full sequence is known. Each distinct proteoform is then quantified accordingly, and the quantity of different proteoforms belonging to a same toxin family should be added up to represent the relative composition of the protein family in a whole venom.
(ii) Protein abundances should be measured *quantitatively* and not *qualitatively*, i.e., the expressed percentage should indicate the relative amount instead of relative number of proteins identified. The protein abundances are estimated based on the tandem MS (MS/MS) spectra derived from individual protein in terms of its peptide count or spectral intensity, with or without integrating additional parameters, such as chromatogram peak area and/or gel intensity (for studies involving protein decomplexation prior to MS analysis).
(iii) There should be inclusivity of a label-free protein quantitation approach in the absence of model organism’s genome or transcriptome. Even with the presence of a model organism’s database, one should realize that an individual specimen may not capture the entire genomic (and therefore proteomic) diversity of a species, especially so when it has many populations in which genetic variability is anticipated. The proteomic algorithms for protein search and quantitation should be optimized using a more inclusive database that incorporates sequence information from phylogenetically related snakes and the species itself where available, instead of restricting the search to only one single species and worse still, a single specimen.
(iv) Both top-down and bottom-up sequencing techniques have pros and cons. Acknowledging the differences and understanding the limitations associated with each approach will help to reduce conflicting views among researchers. Both approaches may complement each other, although the top-down method is apparently more attractive as it allows characterization of intact proteins and PTMs. It is, however, high in cost, technically difficult, less established in the venomic field, and is unavailable in most laboratories. Adopting the middle-down sequencing technique may be a promising solution in this regard.

### 4.3. Interpretation and Application of Findings

Findings from venomics are proven useful in studying the evolution of venomous species, improving the treatment of envenomation, and biodiscovery of therapeutics. However, the comprehensiveness of venomic data is always questionable, and this is obvious in proteomics as the proteins come with very different sizes, hydrophobicities, glycosylations, foldings, isoelectric points, etc., that challenge the differential detectability of MS and quantitation of protein abundances. The problem is perhaps particularly severe for top-down proteomics in which whole native proteins are to be analyzed. The bottom-up and middle-down proteomics, on the other hand, deal with truncated peptides that come with a much lower chemical diversity. Some of these peptides are assumed to be detected confidently as a proxy for the respective proteins, notwithstanding the fact that post-translational modifications, which somehow contribute to venom protein complexity, could be missed. Regardless, the comprehensiveness of a venom proteome can always be questioned, as what is unseen or unknown to the researchers simply does not mean to be absent even when there is a reference model organism’s database. In this pan-genome era, it should be understood that no one single database can stand still to claim the comprehensiveness of all sequences, be it DNA, RNA, or protein of a particular species. Very often, there are even multiple genomes and transcriptomes from the same species, challenging the concept of a reference database of model organism. Simply put, one individual snake specimen can no longer capture the genomic (and therefore proteomic) diversity of a population or species. Furthermore, with the ever-advancing sequencing and bioinformatic techniques, assemblies are deepened, gene predictions are improved, and more versions of genomes are made available from time to time.

**Figure 7:**
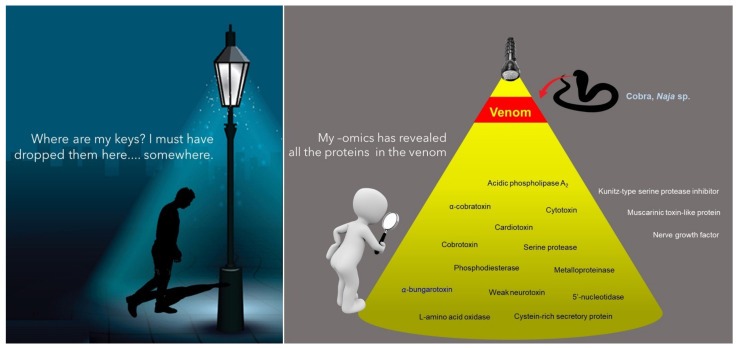
Left panel: The streetlight effect illustrated by a man searching for lost keys where the light is better. This is a metaphor of cognitive availability bias or observational bias. Right panel: The flashlight, symbolizing the venomic tool, sheds light on the venom composition (proteome) of a cobra venom. The metaphoric cartoon shows how data could be misinterpreted: (1) The real dataset is under-represented when whatever (genes and proteins) *revealed under the light* are concluded as all that a species/specimen has, while ignoring what possibly *lies beyond the edge of light*. In this case, the Kunitz-type serine protease inhibitor, muscarinic toxin-like proteins, and nerve-growth factor, somehow undetected, were simply left out. (2) The dataset is over-interpreted when enormous data *shined by the light* are not carefully filtered and validated to represent the species/specimen studied. In this example of a cobra’s venomics, the detection of alpha-bungarotoxin, a krait-specific three-finger toxin should have raised suspicion if it is a false identification.

The question is, how far can we push the limits in venomics to effectively capitalize on the pan-genomic data, so as to claim that one’s study is more superior than the other in terms of “comprehensiveness”? It is virtually impossible to be sure. Yet, it is not uncommon to come across ideas that fixate on particular data generated at a point of time, making inferential conclusions of whether or not a gene (and its protein) is unique to, or, present/absent in a species. On the other hand, it is also impossible to pre-empt poorly filtered data from being reported or deposited in the public database. Hence, in the interpretation of venomic findings, it would be wise to take it with a pinch of salt so as not to fall into the “streetlight effect”, which symbolizes cognitive availability bias. As depicted in Figure 7, the man was looking for his lost keys right under the streetlight, believing it was where he dropped them simply because there was light to allow the search. This is a metaphor of observational bias, which, unfortunately, is also a rather prevalent issue in science, including the -omics field, where researchers might resort to believing that all that was seen (data) represent all there are for a species, while ignoring the limits of proxy, surrogate measure, and the tool (methodology) used in the study. The outcome is inevitably a biased one that sends the study off track when the researcher looks for answers *where the light is better* rather than where the truth is more likely to lie. As it is, the *comprehensiveness* of venom constituents could be under-represented as a significant number of genes and proteins went undetected (due to technical limitations or inherent variation exhibited by a specimen), or over-interpreted with artifacts when data fed by MS were not carefully scrutinized, filtered and validated. Looping back to the streetlight metaphor, one should therefore acknowledge what potentially lies outside the edge of light (*Did I drop my keys in the dark?*), and one should be vigilant enough to tell apart the real from fake found under the light (*Which keys are mine, or rather which are not mine?*). Asking these analogous questions may help to reduce one’s tendency to misinterpret the -omic data with intellectual shortcuts (in this case, snake venom proteomics is referred to), while humbly welcoming new insights from future studies built on improved technologies. After all, one can only trust that science is self-correcting, and in good faith the field shall continue to benefit from constantly proving and disproving established knowledge with new evidence. In the author’s opinion, the venomic journey is a never-ending quest, and present-day discoveries are far from the absolute truth—they are merely steps and paths that lead us closer and closer to the truth.

## 5. Conclusions and Future Perspectives

The venom-producing phenotype in animals, best illustrated by venomous snakes, facilitated the shift from a mechanical to a more efficient biochemical method of predation and defense. Understanding the complexity and diversity of venom is crucial for the improvement of envenoming treatment, evolutionary studies, conservation, and biodiscovery. The transcriptomics and genomics of venomous snakes are greatly advanced by the advent of next-generation sequencing techniques, whereas the progress in venom proteomics is largely driven by advancement in mass spectrometry techniques and expanding databases alongside. Various preparative methods are available to optimize the proteome profiling; these, broadly, are divided into non-decomplexation and decomplexation methods, where the latter incorporates protein separation techniques, such as chromatography and gel electrophoresis prior to mass spectrometry analysis. Conventionally, the venom proteins are digested enzymatically, followed by in-silico sequencing of the many short fragmentary peptides before identification based on the best homology match to proteins in databases (bottom-up approach). A more recently tested method is to sequence the whole protein intact without enzymatic digestion (top-down approach). While both approaches have pros and cons, researchers in the future should attempt to apply the alternative “middle-path” method, i.e., the middle-down sequencing technique where protein digestion is modified so that peptides with moderate length are produced for sequencing.

Undeniably, proteomic methodologies or protocols adopted in snake venomics varied widely across studies, resulting in a massive amount of information that is sometimes confusing with questionable validity. The identification of protein species (or proteforms) relies on the resolution capacity of a spectrometer and its sequencing accuracy, while the quantitation of protein is much more influenced by researchers’ definition of what an abundance is, and the calculation method to obtain the abundance values. This is often the most debated part of a snake venom proteomic study, and truly needs to be reconciled with a consensus among experts. While the strength of venomics is well known, one should be aware of its weakness and not to fall into the cognitive bias akin to the streetlight effect. Two extremes of data misinterpretation are common: At one end, the researcher inflexibly believes in only what one dataset presents and argues against the presence of genes or proteins not hitherto detected, thus oversimplifying the venom’s diversity, functionality, and medical impact. At the other end, the researcher is misled by the presence of invalid genes or proteins identified in the sample, overrating the venom composition and biological implications.

Another challenge faced in venomic research is difficulty related to sampling (venom and tissue). Techniques for sample collection and handling are fairly well established at present, with equipment and reagents that can efficiently minimize sample degradation and contamination upon collection. The difficulty, in fact, is commonly due to inflexible rules and regulations pertaining to the collection of wildlife specimens, including their biological derivatives, and transfer of materials across borders. Awareness of the importance of the work should be promoted to the government and public for improved policy. International organizations, such as the WHO, and global teams related to snakebite initiatives, as well as international funders, can provide fair recognition and support to more expert representatives from various regions of the world. Inclusive and genuine collaboration across borders is the way forward.

Moving forward, the field of snake venomics needs constant improvement of methodology, updating of the database, and expansion of study subjects to include lesser-known species as well as those with geographically varied venom phenotypes. A standardized method that is “one-size-fits-all” is not realistic in venomics; hence, the pros and cons of various methods should be acknowledged and diverging views among different schools of researchers ought to be addressed and reconciled. Snake venomics, in the next decade, should be able to answer the many fundamental questions pertaining to venom evolution and toxin biology, and successfully contribute toward the WHO’s effort in reducing the mortality and morbidity of snakebite envenoming by the half in 2030.

## Figures and Tables

**Table 1:** Comparison of decomplexation and non-decomplexation venom proteomics.

	Decomplexation	Non-Decomplexation
Sample requirement	Moderate to large amount especially if chromatography is involved, typically in milligrams of protein.	Minute amount, typically in as low as micrograms of protein.
Techniques	Protein separation methods applying chromatography, e.g., reverse-phase/ion-exchange/size-exclusion HPLC, and gel electrophoresis techniques (1D or 2D).	Proteins in venom sample are subjected to mass spectrometry (including the preparative work of protein digestion) without prior biochemical separation *.
Downstream experiment	Proteins eluted from chromatography can be readily collected for further purification and characterization.	Limited.
Advantages	Provides additional information regarding protein characteristics, e.g., hydrophobicity, pI, and molecular size. Further downstream studies, e.g., toxin-specific neutralization and antivenomics, are possible.	Technically less demanding. Time-saving and profiling can be achieved fast with fewer resources. Useful when venom sample is limited.
Disadvantages	Laborious and time-consuming. Large amount of sample and more resources are required.	Limited information of protein characteristics.
Examples	HPLC [94,95] HPLC and 1D SDS-PAGE [29,96] 1D SDS-PAGE [83,97] 2D SDS-PAGE [81,98]	[49,92,93,99]

* In top-down proteomics, while the proteins are not subjected to digestion prior to mass spectrometry (MS) analysis, they are fractionated whole by nano-scale liquid chromatography coupled to tandem MS. HPLC: High-performance liquid chromatography; SDS-PAGE: Sodium dodecyl sulfate-polyacrylamide gel electrophoresis.

**Table 2:** Comparison of bottom-up and top-down proteomics used for protein identification in snake venomics.

	Bottom-Up	Top-Down
Protein truncation	Yes, achieved by proteolytic digestion with enzymes, e.g., trypsin and chymotrypsin. Commonly performed as in-solution or in-gel digestion.	Venom proteins are not subjected to proteolytic digestion.
Protein/peptide size	Peptides of ~7–20 amino acid residues (0.8–2 kDa) are analyzed.	The intact protein is analyzed whole.
Ionization and Fragmentation	Peptides from proteolytic cleavage are ionized by ESI/MALDI techniques.	Intact protein is fragmented in the mass spectrometer. Fragmentation is accomplished by ECD or ETD.
Advantages	Technique is mature, commonly used, and widely available. Less sophisticated instrumentation and expertise are required. High-resolution separations can be achieved.	Technique avoids time-consuming protein digestion (typically overnight). Full amino acid sequence can be recovered. Protein isoforms can be determined.PTMs can be located and characterized.
Disadvantages	A low percentage coverage of the amino acid sequence. Information of PTMs is limited.	Instrumentation is expensive and operation is technically sophisticated. Not commonly available. The favored dissociation techniques (ECT, ETC) are less efficient. Resolution is low, especially for high molecular weight proteins.

**Table 3:** Snake genomes available to date as deposited in the public database.

No.	Date of Submission	Common Name	Scientific Name	Family	Sex	Assembly Type	Genome Representation	Notes
1	15/09/2013	Burmese python	*Python bivittatus*	Pythonidae	Female	Scaffold	Full	GCA_000186305.2
2	11/12/2013	King Cobra	*Ophiophagus hannah*	Elapidae	Male	Scaffold	Full	GCA_000516915.1
3	01/08/2014	Southwestern Speckled Rattlesnake	*Crotalus pyrrhus*	Viperidae	Female	Scaffold	Full	GCA_000737285.1
4	10/12/2014	European Adder	*Vipera berus berus*	Viperidae	Female	Scaffold	Full	GCA_000800605.1
5	26/06/2015	Common Garter Snake	*Thamnophis sirtalis*	Colubridae	Female	Scaffold	Full	GCA_001077635.2
6	22/01/2016	Brown Spotted Pitviper; Taiwanese Habu	*Protobothrops mucrosquamatus*	Viperidae	Not stated	Scaffold	Full	GCA_001527695.3
7	21/04/2016	Timber Rattlesnake	*Crotalus horridus*	Viperidae	Female	Scaffold	Full	GCA_001625485.1
8	02/08/2018	Okinawa Habu	*Protobothrops flavoviridis*	Viperidae	Female	Scaffold	Full	GCA_003402635.1
9	05/09/2018	Xizang Hot-spring Keel-back	*Thermophis baileyi* Ecotype: Yangbajing	Colubridae	Female	Scaffold	Full	GCA_003457575.1
10	24/09/2018	Eastern Brown Snake	*Pseudonaja textilis*	Elapidae	Not stated	Scaffold	Full	GCA_900518735.1
11	24/09/2018	Mainland Tiger Snake	*Notechis scutatus*	Elapidae	Not stated	Scaffold	Full	GCA_900518725.1
12	08/01/2019	Prairie Rattlesnake	*Crotalus viridis viridis*	Viperidae	Male	Chromosome	Full	GCA_003400415.2
13	09/01/2019	Ijima’s Turtleheaded Sea Snake	*Emydocephalus ijimae*	Elapidae	Not stated	Scaffold	Full	GCA_004319985.1
14	09/01/2019	Yellow-Lipped Sea Krait	*Laticauda colubrina*	Elapidae	Not stated	Scaffold	Full	GCA_004320045.1
15	09/01/2019	Blue-ringed Sea Krait	*Laticauda laticaudata*	Elapidae	Not stated	Scaffold	Full	GCA_004320025.1
16	15/01/2019	Asian Annulated Sea Snake	*Hydrophis cyanocinctus*	Elapidae	Not stated	Scaffold	Full	GCA_004023725.1
17	15/01/2019 (latest)	Hardwick’s Sea Snake	*Hydrophis hardwickii*	Elapidae	Not stated	Scaffold	Full	GCA_004023765.1
18	13/02/2019	Slender-necked Sea Snake	*Hydrophis melanocephalus*	Elapidae	Not stated	Scaffold		GCA_004320005.1
19	11/12/2019	Indian Cobra	*Naja naja*	Elapidae	male	Chromosome	Full	GCA_009733165.1
20	19/12/2019	Western Terrestrial Garter Snake	*Thamnophis elegans*	Colubridae	Female	Type: alternate-pseudohaplotype Level: Scaffold	Full	GCA_009769695.1
21	23/12/2019	Western Terrestrial Garter Snake	*Thamnophis elegans*	Colubridae	Female	Assembly type: haploid (principal pseudohaplotype of diploid) Assembly level: Chromosome	Full	GCA_009769535.1
22	13/04/2020	Corn Snake	*Pantherophis guttatus*	Colubridae	Male	Scaffold	Full	GCA_001185365.2
23	22/04/2020	Western Rat Snake	*Pantherophis obsoletus*	Colubridae	Female	Scaffold	Full	GCA_012654085.1
24	22/04/2020	Dhaman; Oriental Ratsnake	*Ptyas mucosa*	Colubridae	Female	Scaffold	Full	GCA_012654045.1
25	04/11/2020 (latest)	Yellow-Lipped Sea Krait	*Laticauda colubrina*	Elapidae	Not stated	Scaffold	Full	GCA_015471245.1
26	22/11/2020	Eastern Brown Snake	*Pseudonaja textilis*	Elapidae	Not stated	Scaffold	Full	GCA_900608585.1
27	22/11/2020 (latest)	Mainland Tiger Snake	*Notechis scutatus*	Elapidae	Not stated	Scaffold	Full	GCA_900608555.1
28	06/01/2021	Tiger Rattlesnake	*Crotalus tigris* Infraspecific name: Ecotype: Arizon	Viperidae	Not stated	Contig	Full	GCA_016545835.1
29	01/04/2021	Mud Snake	*Myanophis thanlyinensis*	Homalopsidae	Male	Scaffold	Full	GCA_017656035.1
30	19/04/2021	Indian Cobra	*Naja naja*	Elapidae	Female	Scaffold	Full	GCA_018093825.1
31	2021/05/11/05/2021	Jararaca	*Bothrops jararaca*	Viperidae	Female	Scaffold	Full	GCA_018340635.1
32	25/05/2021	Eastern Diamondback Rattlesnake	*Crotalus adamanteus*	Viperidae	Female	Scaffold	Full	GCA_018446365.1
33	06/08/2021	Golden Tree Snake	*Chrysopelea ornata*	Colubridae	Not stated	Scaffold	Full	GCA_019457695.1
34	09/08/2021	Shaw’s Sea Snake	*Hydrophis curtus*	Elapidae	Male	Chromosome	Full	GCA_019472885.1
35	09/08/2021	Annulated Sea Snake	*Hydrophis cyanocinctus*	Elapidae	Male	Chromosome	Full	GCA_019473425.1
36	18/08/2021	Gopher Snake	*Pituophis catenifer pumilus* Infraspecific name: Ecotype: Santa Cruz Island	Colubridae	Female	Scaffold	Full	GCA_019677565.1
37	23/02/2022	Prong-snouted blind snake	*A* *nilios bituberculatus*	Typhlopidae	Not stated	Sca	Full	GCA_022379055.1
38	15/03/2022	Glossy snake	*Arizona elegans* Infraspecific name: Ecotype: subspecies *occidentalis*	Colubridae	Not stated	Scaffold (alternate-pseudohaplotype)	Full	GCA_022578425.1
39	15/03/2022	Glossy snake	*Arizona elegans* Infraspecific name: Ecotype: subspecies *occidentalis*	Colubridae	Not stated	Scaffold (haploid, principal pseudohaplotype of diploid)	Full	GCA_022577455.1

**Table 4:** Comparison of toxins identified in *Trimeresurus puniceus* venoms by protein families, subtypes, and relative abundances.

Protein Family/Subtype ^a^	Accession No. ^b,c^	Relative Protein Abundance ^d^ (%)	
		Method 1	Method 2
**Snake Venom Serine Protease (15)**		**19.03**	**25.42**
Alpha-fibrinogenase albofibrase	P0CJ41	3.74	1.69
Alpha-fibrinogenase shedaoenase	Q6T5L0	1.20	1.69
Beta-fibrinogenase mucrofibrase-2	Q91508	1.08	1.69
Snake venom serine protease 1	Unigene42520_TWM	1.46	1.69
Snake venom serine protease 2	CL403.contig2_TWM	0.58	1.69
Snake venom serine protease 2A homolog	O13060	1.01	1.69
Snake venom serine protease 2C	O13062	0.53	1.69
Snake venom serine protease KN14	Q71QH9	1.70	1.69
Snake venom serine protease KN8	Q71QH5	0.87	1.67
Snake venom serine protease pallase	O93421	1.34	1.69
Snake venom serine protease salmonase	Q9PTL3	1.16	1.69
Thrombin-like enzyme 1	A7LAC6	0.80	1.69
Thrombin-like enzyme 2	A7LAC7	1.19	1.69
Thrombin-like enzyme calobin-1	Q91053	0.65	1.69
Venom plasminogen activator TSV-PA	Q91516	1.71	1.69
**Snake Venom Metalloproteinase (11)**		**17.29**	**18.64**
***P-II SVMP***			
Zinc metalloproteinase/disintegrin	Unigene5053_TWM	0.34	1.69
Zinc metalloproteinase homolog-disintegrin albolatin	P0C6B6	0.92	1.69
***P-III SVMP***			
Zinc metalloproteinase/disintegrin	P0C6E8	1.30	1.69
Zinc metalloproteinase-disintegrin-like ACLD	CL1397.contig5_TWM	1.73	1.69
Zinc metalloproteinase-disintegrin-like ACLD	O42138	0.17	1.69
Zinc metalloproteinase-disintegrin-like HF3	Q98UF9	1.81	1.69
Zinc metalloproteinase-disintegrin-like stejnihagin-A	Q3HTN1	1.70	1.69
Zinc metalloproteinase-disintegrin-like stejnihagin-B	Q3HTN2	0.57	1.69
Zinc metalloproteinase-disintegrin-like TSV-DM	J3RYA3	0.16	1.69
Zinc metalloproteinase-disintegrin-like VMP-III	CL1397.contig1_TWM	8.41	1.69
Zinc metalloproteinase-disintegrin-like VMP-III	C9E1S0	0.18	1.69
**Disintegrin (2)**		**15.82**	**3.39**
Disintegrin albolabrin	P62384	4.74	1.69
Disintegrin trigramin-gamma	P62383	11.08	1.69
**C-type Lectin (9)**		**8.92**	**15.25**
C-type lectin 6	Unigene46336_TWM	1.01	1.69
C-type lectin TsL	Q9YGP1	1.85	1.69
Snaclec alboaggregin-D subunit alpha	P0DM38	0.88	1.69
Snaclec clone 2100755	Q8JIV8	0.85	1.69
Snaclec coagulation factor IX/factor X-binding protein subunit A	Q71RR4	0.70	1.69
Snaclec convulxin subunit alpha	Unigene46337_TWM	0.35	1.69
Snaclec purpureotin subunit alpha	P0DJL2	1.48	1.69
Snaclec purpureotin subunit beta	P0DJL3	1.27	1.69
Snaclec stejaggregin-A subunit alpha	CL746.contig2_TWM	0.53	1.69
**Phospholipase A_2_ (7)**		**27.54**	**11.86**
Acidic phospholipase A_2_	P20249	2.54	1.69
Acidic phospholipase A_2_ 6	P70088	2.77	1.69
Acidic phospholipase A_2_ Tpu-E6c	P0DJP4	4.77	1.69
Basic phospholipase A_2_ daboxin P	C0HK16	1.18	1.69
Basic phospholipase A_2_ homolog Tpu-K49a	Q2YHJ9	7.50	1.69
Basic phospholipase A_2_ homolog Tpu-K49b	Q2YHJ8	4.45	1.69
Basic phospholipase A_2_ Tpu-G6D49	Q2YHJ7	4.33	1.69
**Cysteine-rich Secretory Protein (3)**		**1.69**	**5.08**
Cysteine-rich secretory protein	P60623	0.91	1.69
Cysteine-rich secretory protein	Unigene30615_TWM	0.26	1.69
Cysteine-rich secretory protein triflin	Q8JI39	0.52	1.69
**L-Amino Acid Oxidase (4)**		**4.82**	**6.78**
L-amino-acid oxidase	B0VXW0	0.66	1.69
L-amino-acid oxidase	Q6WP39	1.72	1.69
L-amino-acid oxidase	Q90W54	0.87	1.69
L-amino-acid oxidase	Unigene40029_TWM	1.57	1.69
**Snake Venom Phosphodiesterase (3)**		**2.27**	**5.08**
Phosphodiesterase	Unigene5177_TWM	0.65	1.69
Venom phosphodiesterase 1	J3SEZ3	0.71	1.69
Venom phosphodiesterase 2	J3SBP3	0.90	1.69
**Snake Venom Vascular Endothelial Growth Factor (1)**		**2.49**	**1.69**
Snake venom vascular endothelial growth factor toxin	Unigene25068_TWM	1.54	1.69
**Snake Venom 5′-Nucleotidase (2)**		**0.71**	**3.39**
Snake venom 5′-nucleotidase	Unigene721_TWM	0.38	1.69
Snake venom 5′-nucleotidase	B6EWW8	0.33	1.69
**Snake Venom Nerve Growth Factor (1)**		**0.18**	**1.69**
Nerve growth factor	CL2590.contig1_TWM	0.18	1.69
**Phospholipase B (1)**		**0.18**	**1.69**
Phospholipase B	Unigene25350_TWM	0.18	1.69
**Total number of proteins: 59**	**Total protein abundance (%)**	**100**	**100**

^a,b^ Protein identification, accession numbers, and corresponding species were derived from databases based on best homology match. Number in parenthesis: Total number of distinct proteins matched for individual protein/toxin family. ^c^ Accession numbers with suffix “_TWM” were based on an in-house transcript-database specific for *Trimeresurus wiroti* (Malaysia). ^d^ Relative abundance is calculated by two different methods: (1) Method 1: By incorporating the relative spectral intensity (of non-redundant peptides belonging to individual protein) with the area under the curve of chromatographic fraction. (2) Method 2: By dividing the number of individual proteins by the total number of all proteins identified in the venom. In this example, the total number of proteins was 59, which also served as the denominator. Proteomic data for the species and Method 1 were derived from the author’s previously published work [47].

## References

[1] Mackessy P.S., Mackessy P.S. (2021). Reptile Venoms and Toxins: Unlimited Opportunities for Basic and Applied Research. Handbook of Venoms and Toxins of Reptiles.

[2] Nelsen D.R., Nisani Z., Cooper A.M., Fox G.A., Gren E.C.K., Corbit A.G., Hayes W.K. (2014). Poisons, toxungens, and venoms: Redefining and classifying toxic biological secretions and the organisms that employ them. Biol. Rev..

[3] Wong E.S.W., Morgenstern D., Mofiz E., Gombert S., Morris K.M., Temple-Smith P., Renfree M., Whittington C., King G., Warren W.C. (2012). Proteomics and Deep Sequencing Comparison of Seasonally Active Venom Glands in the Platypus Reveals Novel Venom Peptides and Distinct Expression Profiles. Mol. Cell. Proteom..

[4] Post D.C., Jeanne R.L. (1983). Venom Source of a sex pheromone in the social wasp *Polistes fuscatus* (Hymenoptera: Vespidae). J. Chem. Ecol..

[5] LeBrun E.G., Diebold P.J., Orr M.R., Gilbert L.E. (2015). Widespread Chemical Detoxification of Alkaloid Venom by Formicine Ants. J. Chem. Ecol..

[6] Saviola A.J., Chiszar D., Busch C., Mackessy S.P. (2013). Molecular basis for prey relocation in viperid snakes. BMC Biol..

[7] Gutiérrez J.M., Calvete J., Habib A.G., Harrison R.A., Williams D., Warrell D.A. (2017). Correction: Snakebite envenoming. Nat. Rev. Dis. Primers.

[8] Fry B.G. (2018). Snakebite: When the Human Touch Becomes a Bad Touch. Toxins.

[9] King G.F. (2011). Venoms as a platform for human drugs: Translating toxins into therapeutics. Expert Opin. Biol. Ther..

[10] Kalita B., Saviola A.J., Mukherjee A.K. (2021). From venom to drugs: A review and critical analysis of Indian snake venom toxins envisaged as anticancer drug prototypes. Drug Discov. Today.

[11] Mackessy S.P. (2010). Evolutionary trends in venom composition in the Western Rattlesnakes (*Crotalus viridis* sensu lato): Toxicity vs. tenderizers. Toxicon.

[12] Fry B.G., Vidal N., Norman J.A., Vonk F.J., Scheib H., Ramjan S.F.R., Kuruppu S., Fung K., Hedges S.B., Richardson M.K. (2005). Early evolution of the venom system in lizards and snakes. Nature.

[13] Fry B.G., Casewell N.R., Wüster W., Vidal N., Young B., Jackson T.N.W. (2012). The structural and functional diversification of the Toxicofera reptile venom system. Toxicon.

[14] Casewell N.R., Wüster W., Vonk F.J., Harrison R.A., Fry B.G. (2013). Complex cocktails: The evolutionary novelty of venoms. Trends Ecol. Evol..

[15] Weinstein S.A., Keyler D.E., White J. (2012). Replies to Fry et al. (Toxicon 2012, 60/4, 434–448). Part A. Analyses of squamate reptile oral glands and their products: A call for caution in formal assignment of terminology designating biological function. Toxicon.

[16] Kardong K.V. (2012). Replies to Fry et al. (Toxicon 2012, 60/4, 434–448). Part B. Properties and biological roles of squamate oral products: The “venomous lifestyle” and preadaptation. Toxicon.

[17] Reyes-Velasco J., Card D.C., Andrew A.L., Shaney K.J., Adams R.H., Schield D.R., Casewell N., Mackessy S., Castoe T.A. (2014). Expression of Venom Gene Homologs in Diverse Python Tissues Suggests a New Model for the Evolution of Snake Venom. Mol. Biol. Evol..

[18] Hargreaves A.D., Swain M.T., Logan D.W., Mulley J.F. (2014). Testing the Toxicofera: Comparative transcriptomics casts doubt on the single, early evolution of the reptile venom system. Toxicon.

[19] Augusto-De-Oliveira C., Stuginski D.R., Kitano E.S., Andrade-Silva D., Liberato T., Fukushima I., Serrano S.M.T., Zelanis A. (2016). Dynamic Rearrangement in Snake Venom Gland Proteome: Insights into Bothrops jararaca Intraspecific Venom Variation. J. Proteome Res..

[20] Casewell N.R., Jackson T.N.W., Laustsen A.H., Sunagar K. (2020). Causes and Consequences of Snake Venom Variation. Trends Pharmacol. Sci..

[21] Tan K.Y., Tan C.H., Chanhome L., Tan N.H. (2017). Comparative venom gland transcriptomics of *Naja kaouthia* (monocled cobra) from Malaysia and Thailand: Elucidating geographical venom variation and insights into sequence novelty. PeerJ.

[22] Calvete J.J., Sanz L., Angulo Y., Lomonte B., Gutierrez J.M. (2009). Venoms, venomics, antivenomics. FEBS Lett..

[23] Calvete J.J. (2013). Snake venomics: From the inventory of toxins to biology. Toxicon.

[24] Lomonte B., Calvete J.J. (2017). Strategies in ‘snake venomics’ aiming at an integrative view of compositional, functional, and immunological characteristics of venoms. J. Venom. Anim. Toxins Incl. Trop. Dis..

[25] Wilson D., Daly N.L. (2018). Venomics: A Mini-Review. High-Throughput.

[26] Juárez P., Sanz L., Calvete J.J. (2004). Snake venomics: Characterization of protein families in *Sistrurus barbouri* venom by cysteine mapping, N-terminal sequencing, and tandem mass spectrometry analysis. Proteomics.

[27] Calvete J.J., Sanz L., Mora-Obando D., Lomonte B., Tanaka-Azevedo A.M., de Morais-Zani K., Sant’Anna S.S., Caldeira C.A. (2021). What’s in a mass?. Biochem. Soc. Trans..

[28] Tan K.Y., Liew J.L., Tan N.H., Quah E.S., Ismail A.K., Tan C.H. (2018). Unlocking the secrets of banded coral snake (*Calliophis intestinalis*, Malaysia): A venom with proteome novelty, low toxicity and distinct antigenicity. J. Proteom..

[29] Tan K.Y., Tan C.H., Fung S.Y., Tan N.H. (2015). Venomics, lethality and neutralization of *Naja kaouthia* (monocled cobra) venoms from three different geographical regions of Southeast Asia. J. Proteom..

[30] Gutiérrez J.M., Lomonte B., León G., Alape-Girón A., Flores-Díaz M., Sanz L., Angulo Y., Calvete J. (2009). Snake venomics and antivenomics: Proteomic tools in the design and control of antivenoms for the treatment of snakebite envenoming. J. Proteom..

[31] Vetter I., Davis J.L., Rash L.D., Anangi R., Mobli M., Alewood P.F., Lewis R.J., King G.F. (2011). Venomics: A new paradigm for natural products-based drug discovery. Amino Acids.

[32] Fox J.W., Serrano S.M.T. (2008). Exploring snake venom proteomes: Multifaceted analyses for complex toxin mixtures. Proteomics.

[33] Calvete J.J., Petras D., Calderón-Celis F., Lomonte B., Encinar J.R., Sanz-Medel A. (2017). Protein-species quantitative venomics: Looking through a crystal ball. J. Venom. Anim. Toxins Incl. Trop. Dis..

[34] Calvete J.J., Lomonte B., Saviola A.J., Bonilla F., Sasa M., Williams D.J., Undheim E.A.B., Sunagar K., Jackson T.N.W. (2021). Mutual enlightenment: A toolbox of concepts and methods for integrating evolutionary and clinical toxinology via snake venomics and the contextual stance. Toxicon X.

[35] Au-Garb J.E. (2014). Extraction of Venom and Venom Gland Microdissections from Spiders for Proteomic and Transcriptomic Analyses. JoVE.

[36] Ward M.J., Ellsworth S.A., Rokyta D.R. (2018). Venom-gland transcriptomics and venom proteomics of the Hentz striped scorpion (*Centruroides hentzi*; Buthidae) reveal high toxin diversity in a harmless member of a lethal family. Toxicon.

[37] Jouiaei M., Casewell N.R., Yanagihara A.A., Nouwens A., Cribb B.W., Whitehead D., Jackson T.N.W., Ali S.A., Wagstaff S.C., Koludarov I. (2015). Firing the Sting: Chemically Induced Discharge of Cnidae Reveals Novel Proteins and Peptides from Box Jellyfish (*Chironex fleckeri*) Venom. Toxins.

[38] Tan C.H., Tan K.Y., Yap M.K.K., Tan N.H. (2017). Venomics of Tropidolaemus wagleri, the sexually dimorphic temple pit viper: Unveiling a deeply conserved atypical toxin arsenal. Sci. Rep..

[39] Amorim F.G., Costa T.R., Baiwir D., De Pauw E., Quinton L., Sampaio S.V. (2018). Proteopeptidomic, Functional and Immuno-reactivity Characterization of Bothrops moojeni Snake Venom: Influence of Snake Gender on Venom Composition. Toxins.

[40] Menezes M.C., Furtado M.F., Travaglia-Cardoso S.R., Camargo A.C.M., Serrano S.M.T. (2006). Sex-based individual variation of snake venom proteome among eighteen *Bothrops jararaca* siblings. Toxicon.

[41] Zelanis A., Tashima A.K., Rocha M.M.T., Furtado M.F., Camargo A.C.M., Ho P.L., Serrano S.M.T. (2010). Analysis of the Ontogenetic Variation in the Venom Proteome/Peptidome of *Bothrops jararaca* Reveals Different Strategies to Deal with Prey. J. Proteome Res..

[42] Wray K.P., Margres M.J., Seavy M., Rokyta D.R. (2015). Early significant ontogenetic changes in snake venoms. Toxicon.

[43] Arbuckle K., Gopalakrishnakone P., Malhotra A. (2017). Evolutionary context of venom in animals. Evolution of Venomous Animals and Their Toxins.

[44] Pla D., Sanz L., Quesada-Bernat S., Villalta M., Baal J., Chowdhury M.A.W., León G., Gutiérrez J.M., Kuch U., Calvete J.J. (2019). Phylovenomics of *Daboia russelii* across the Indian subcontinent. Bioactivities and comparative in vivo neutralization and in vitro third-generation antivenomics of antivenoms against venoms from India, Bangladesh and Sri Lanka. J. Proteom..

[45] Faisal T., Tan K.Y., Sim S.M., Quraishi N., Tan N.H., Tan C.H. (2018). Proteomics, functional characterization and antivenom neutralization of the venom of Pakistani Russell’s viper (*Daboia russelii*) from the wild. J. Proteom..

[46] Patra A., Mukherjee A.K. (2020). Proteomic Analysis of Sri Lanka *Echis carinatus* Venom: Immunological Cross-Reactivity and Enzyme Neutralization Potency of Indian Polyantivenom. J. Proteome Res..

[47] Lee L.P., Tan K.Y., Tan C.H. (2021). Snake venom proteomics and antivenomics of two Sundaic lance-headed pit vipers: *Trimeresurus wiroti* (Malaysia) and *Trimeresurus puniceus* (Indonesia). Comp. Biochem. Physiol. Part D Genom. Proteom..

[48] Liew J.L., Tan N.H., Tan C.H. (2020). Proteomics and preclinical antivenom neutralization of the mangrove pit viper (*Trimeresurus purpureomaculatus*, Malaysia) and white-lipped pit viper (*Trimeresurus albolabris*, Thailand) venoms. Acta Trop..

[49] Tan C.H., Palasuberniam P., Tan K.Y. (2021). Snake Venom Proteomics, Immunoreactivity and Toxicity Neutralization Studies for the Asiatic Mountain Pit Vipers, *Ovophis convictus*, *Ovophis tonkinensis*, and Hime Habu, *Ovophis okinavensis*. Toxins.

[50] Silva A., Cristofori-Armstrong B., Rash L.D., Hodgson W.C., Isbister G.K. (2018). Defining the role of post-synaptic α-neurotoxins in paralysis due to snake envenoming in humans. Cell. Mol. Life Sci..

[51] Tan C.H., Wong K.Y., Chong H.P., Tan N.H., Tan K.Y. (2019). Proteomic insights into short neurotoxin-driven, highly neurotoxic venom of Philippine cobra (*Naja philippinensis*) and toxicity correlation of cobra envenomation in Asia. J. Proteom..

[52] Tan N.H., Tan K.Y., Tan C.H., Mackessy S.P. (2021). Snakebite in Southeast Asia: Envenomation and Clinical Management. Handbook of Venoms and Toxins of Reptiles.

[53] Tan N.H., Wong K.Y., Tan C.H. (2017). Venomics of *Naja sputatrix*, the Javan spitting cobra: A short neurotoxin-driven venom needing improved antivenom neutralization. J. Proteom..

[54] Wong K.Y., Tan C.H., Tan N.H. (2016). Venom and Purified Toxins of the Spectacled Cobra (*Naja naja*) from Pakistan: Insights into Toxicity and Antivenom Neutralization. Am. J. Trop. Med. Hyg..

[55] Wüster W., Crookes S., Ineich I., Mané Y., Pook C.E., Trape J.-F., Broadley D.G. (2007). The phylogeny of cobras inferred from mitochondrial DNA sequences: Evolution of venom spitting and the phylogeography of the African spitting cobras (Serpentes: Elapidae: *Naja nigricollis* complex). Mol. Phylogenet. Evol..

[56] Kazemi E., Nazarizadeh M., Fatemizadeh F., Khani A., Kaboli M. (2020). The phylogeny, phylogeography, and diversification history of the westernmost Asian cobra (Serpentes: Elapidae: *Naja oxiana*) in the Trans-Caspian region. Ecol. Evol..

[57] Petras D., Sanz L., Segura A., Herrera M., Villalta M., Solano D., Vargas M., León G., Warrell D.A., Theakston R.D.G. (2011). Snake Venomics of African Spitting Cobras: Toxin Composition and Assessment of Congeneric Cross-Reactivity of the Pan-African EchiTAb-Plus-ICP Antivenom by Antivenomics and Neutralization Approaches. J. Proteome Res..

[58] Lauridsen L.P., Laustsen A.H., Lomonte B., Gutiérrez J.M. (2017). Exploring the venom of the forest cobra snake: Toxicovenomics and antivenom profiling of *Naja melanoleuca*. J. Proteom..

[59] Malih I., Rusmili M.R.A., Tee T.Y., Saile R., Ghalim N., Othman I. (2014). Proteomic analysis of Moroccan cobra *Naja haje legionis* venom using tandem mass spectrometry. J. Proteom..

[60] Kazandjian T.D., Petras D., Robinson S.D., van Thiel J., Greene H.W., Arbuckle K., Barlow A., Carter D.A., Wouters R.M., Whiteley G. (2021). Convergent evolution of pain-inducing defensive venom components in spitting cobras. Science.

[61] Tan C.H., Wong K.Y., Tan N.H., Ng T.S., Tan K.Y. (2019). Distinctive Distribution of Secretory Phospholipases A2 in the Venoms of Afro-Asian Cobras (Subgenus: *Naja*, *Afronaja*, *Boulengerina* and *Uraeus*). Toxins.

[62] Panagides N., Jackson T.N.W., Ikonomopoulou M.P., Arbuckle K., Pretzler R., Yang D.C., Ali S.A., Koludarov I., Dobson J., Sanker B. (2017). How the Cobra Got Its Flesh-Eating Venom: Cytotoxicity as a Defensive Innovation and Its Co-Evolution with Hooding, Aposematic Marking, and Spitting. Toxins.

[63] Tan K.Y., Wong K.Y., Tan N.H., Tan C.H. (2020). Quantitative proteomics of *Naja annulifera* (sub-Saharan snouted cobra) venom and neutralization activities of two antivenoms in Africa. Int. J. Biol. Macromol..

[64] Wong K., Tan K., Tan N., Tan C. (2021). A Neurotoxic Snake Venom without Phospholipase A_2_: Proteomics and Cross-Neutralization of the Venom from Senegalese Cobra, *Naja senegalensis* (Subgenus: *Uraeus*). Toxins.

[65] Palasuberniam P., Chan Y.W., Tan K.Y., Tan C.H. (2021). Snake Venom Proteomics of Samar Cobra (*Naja samarensis*) from the Southern Philippines: Short Alpha-Neurotoxins as the Dominant Lethal Component Weakly Cross-Neutralized by the Philippine Cobra Antivenom. Front. Pharmacol..

[66] Watt G., Laughlin L., Padre L., Tuazon M.L., Theakston R.D.G. (1988). Bites by the Philippine Cobra (*Naja naja philippinensis*): Prominent Neurotoxicity with Minimal Local Signs. Am. J. Trop. Med. Hyg..

[67] Tan C.H., Tan K.Y. (2019). Functional Application of Snake Venom Proteomics in In Vivo Antivenom Assessment. Methods Mol. Biol..

[68] Ratanabanangkoon K., Tan K.Y., Pruksaphon K., Klinpayom C., Gutiérrez J.M., Quraishi N.H., Tan C.H. (2020). A pan-specific antiserum produced by a novel immunization strategy shows a high spectrum of neutralization against neurotoxic snake venoms. Sci. Rep..

[69] Ratanabanangkoon K., Tan K.Y., Eursakun S., Tan C.H., Simsiriwong P., Pamornsakda T., Wiriyarat W., Klinpayom C., Tan N.H. (2016). A Simple and Novel Strategy for the Production of a Pan-specific Antiserum against Elapid Snakes of Asia. PLoS Negl. Trop. Dis..

[70] Wallach V., Wüster W., G Broadley D. (2009). In Praise Of Subgenera: Taxonomic Status Of Cobras Of The Genus *Naja* Laurenti (Serpentes: Elapidae). Zootaxa.

[71] Wong K.Y., Tan C.H., Tan K.Y., Quraishi N.H., Tan N.H. (2018). Elucidating the biogeographical variation of the venom of *Naja naja* (spectacled cobra) from Pakistan through a venom-decomplexing proteomic study. J. Proteom..

[72] Laxme R.R.S., Attarde S., Khochare S., Suranse V., Martin G., Casewell N.R., Whitaker R., Sunagar K. (2021). Biogeographical venom variation in the Indian spectacled cobra (*Naja naja*) underscores the pressing need for pan-India efficacious snakebite therapy. PLoS Negl. Trop. Dis..

[73] Wong K.Y., Tan K.Y., Tan N.H., Gnanathasan C.A., Tan C.H. (2021). Elucidating the Venom Diversity in Sri Lankan Spectacled Cobra (*Naja naja*) through De Novo Venom Gland Transcriptomics, Venom Proteomics and Toxicity Neutralization. Toxins.

[74] Shan L.-L., Gao J.-F., Zhang Y.-X., Shen S.-S., He Y., Wang J., Ma X.-M., Ji X. (2016). Proteomic characterization and comparison of venoms from two elapid snakes (*Bungarus multicinctus* and *Naja atra*) from China. J. Proteom..

[75] Liu C.-C., Chou Y.-S., Chen C.-Y., Liu K.-L., Huang G.-J., Yu J.-S., Wu C.-J., Liaw G.-W., Hsieh C.-H., Chen C.-K. (2020). Pathogenesis of local necrosis induced by *Naja atra* venom: Assessment of the neutralization ability of Taiwanese freeze-dried neurotoxic antivenom in animal models. PLoS Negl. Trop. Dis..

[76] Calvete J.J., Juárez P., Sanz L. (2007). Snake venomics. Strategy and applications. Biol. Mass Spectrom..

[77] Tan C.H., Wong K.Y., Tan K.Y., Tan N.H. (2017). Venom proteome of the yellow-lipped sea krait, *Laticauda colubrina* from Bali: Insights into subvenomic diversity, venom antigenicity and cross-neutralization by antivenom. J. Proteom..

[78] Lingam T.M.C., Tan K.Y., Tan C.H. (2020). Proteomics and antivenom immunoprofiling of Russell’s viper (*Daboia siamensis*) venoms from Thailand and Indonesia. J. Venom. Anim. Toxins Incl. Trop. Dis..

[79] Petras D., Heiss P., Süssmuth R.D., Calvete J.J. (2015). Venom Proteomics of Indonesian King Cobra, *Ophiophagus hannah*: Integrating Top-Down and Bottom-Up Approaches. J. Proteome Res..

[80] Al-Shekhadat R.I., Lopushanskaya K.S., Segura Á., Gutiérrez J.M., Calvete J.J., Pla D. (2019). *Vipera berus berus* Venom from Russia: Venomics, Bioactivities and Preclinical Assessment of Microgen Antivenom. Toxins.

[81] Ali S.A., Baumann K., Jackson T.N., Wood K., Mason S., Undheim E.A., Nouwens A., Koludarov I., Hendrikx I., Jones A. (2013). Proteomic comparison of *Hypnale hypnale* (Hump-Nosed Pit-Viper) and *Calloselasma rhodostoma* (Malayan Pit-Viper) venoms. J. Proteom..

[82] Serrano S.M.T., Shannon J.D., Wang D., Camargo A.C.M., Fox J.W. (2005). A multifaceted analysis of viperid snake venoms by two-dimensional gel electrophoresis: An approach to understanding venom proteomics. Proteomics.

[83] Tan C.H., Fung S.Y., Yap M.K.K., Leong P.K., Liew J.L., Tan N.H. (2016). Unveiling the elusive and exotic: Venomics of the Malayan blue coral snake (*Calliophis bivirgata flaviceps*). J. Proteom..

[84] Li S., Wang J., Zhang X., Ren Y., Wang N., Zhao K., Chen X., Zhao C., Li X., Shao J. (2004). Proteomic characterization of two snake venoms: *Naja naja atra* and *Agkistrodon halys*. Biochem. J..

[85] Calvete J.J. (2014). Next-generation snake venomics: Protein-locus resolution through venom proteome decomplexation. Expert Rev. Proteom..

[86] Tan C.H., Tan K.Y., Tan N.H. (2019). A Protein Decomplexation Strategy in Snake Venom Proteomics. Methods in Molecular Biology.

[87] Tan C.H., Liew J.L., Navanesan S., Sim K.S., Tan N.H., Tan K.Y. (2020). Cytotoxic and anticancer properties of the Malaysian mangrove pit viper (*Trimeresurus purpureomaculatus*) venom and its disintegrin (*purpureomaculin*). J. Venom. Anim. Toxins Incl. Trop. Dis..

[88] Chong H.P., Tan K.Y., Tan C.H. (2020). Cytotoxicity of Snake Venoms and Cytotoxins from Two Southeast Asian Cobras (*Naja sumatrana*, *Naja kaouthia*): Exploration of Anticancer Potential, Selectivity, and Cell Death Mechanism. Front. Mol. Biosci..

[89] Tan C.H., Tan K.Y., Ng T.S., Sim S.M., Tan N.H. (2018). Venom Proteome of Spine-Bellied Sea Snake (*Hydrophis curtus*) from Penang, Malaysia: Toxicity Correlation, Immunoprofiling and Cross-Neutralization by Sea Snake Antivenom. Toxins.

[90] Tan C.H., Tan K.Y., Lim S.E., Tan N.H. (2015). Venomics of the beaked sea snake, *Hydrophis schistosus*: A minimalist toxin arsenal and its cross-neutralization by heterologous antivenoms. J. Proteom..

[91] Lauridsen L.P., Laustsen A.H., Lomonte B., Gutiérrez J.M. (2016). Toxicovenomics and antivenom profiling of the Eastern green mamba snake (*Dendroaspis angusticeps*). J. Proteom..

[92] Aird S.D., Watanabe Y., Villar-Briones A., Roy M.C., Terada K., Mikheyev A.S. (2013). Quantitative high-throughput profiling of snake venom gland transcriptomes and proteomes (*Ovophis okinavensis* and *Protobothrops flavoviridis*). BMC Genom..

[93] Tan C.H., Tan K.Y., Ng T.S., Quah E.S.H., Ismail A.K., Khomvilai S., Sitprija V., Tan N.H. (2019). Venomics of *Trimeresurus* (Popeia) *nebularis*, the Cameron Highlands Pit Viper from Malaysia: Insights into Venom Proteome, Toxicity and Neutralization of Antivenom. Toxins.

[94] Oh A.M.F., Tan K.Y., Tan N.H., Tan C.H. (2021). Proteomics and neutralization of *Bungarus multicinctus* (Many-banded Krait) venom: Intra-specific comparisons between specimens from China and Taiwan. Comp. Biochem. Physiol. Part C Toxicol. Pharmacol..

[95] Kalita B., Patra A., Das A., Mukherjee A.K. (2018). Proteomic Analysis and Immuno-Profiling of Eastern India Russell’s Viper (*Daboia russelii*) Venom: Correlation between RVV Composition and Clinical Manifestations Post RV Bite. J. Proteome Res..

[96] Lomonte B., Tsai W.-C., Ureña-Diaz J.M., Sanz L., Mora-Obando D., Sánchez E.E., Fry B.G., Gutiérrez J.M., Gibbs H.L., Sovic M.G. (2013). Venomics of New World pit vipers: Genus-wide comparisons of venom proteomes across Agkistrodon. J. Proteom..

[97] Tan C.H., Tan K.Y., Fung S.Y., Tan N.H. (2015). Venom-gland transcriptome and venom proteome of the Malaysian king cobra (*Ophiophagus hannah*). BMC Genom..

[98] Ching A.T.C., Leme A.F.P., Zelanis A., Rocha M.M.T., Furtado M.D.F.D., Silva D.A., Trugilho M.R.O., da Rocha S.L.G., Perales J., Ho P.L. (2012). Venomics Profiling of Thamnodynastes strigatus Unveils Matrix Metalloproteinases and Other Novel Proteins Recruited to the Toxin Arsenal of Rear-Fanged Snakes. J. Proteome Res..

[99] Tan K.Y., Tan N.H., Tan C.H. (2018). Venom proteomics and antivenom neutralization for the Chinese eastern Russell’s viper, *Daboia siamensis* from Guangxi and Taiwan. Sci. Rep..

[100] Chapeaurouge A., Reza A., Mackessy S., Carvalho P.C., Valente R.H., Teixeira-Ferreira A., Perales J., Lin Q., Kini M. (2015). Interrogating the Venom of the Viperid Snake *Sistrurus catenatus edwardsii* by a Combined Approach of Electrospray and MALDI Mass Spectrometry. PLoS ONE.

[101] Tan C.H., Tan K.Y., Tan N.H. (2016). Revisiting *Notechis scutatus* venom: On shotgun proteomics and neutralization by the “bivalent” Sea Snake Antivenom. J. Proteom..

[102] Pandeswari P.B., Sabareesh V. (2019). Middle-down approach: A choice to sequence and characterize proteins/proteomes by mass spectrometry. RSC Adv..

[103] Timp W., Timp G. (2020). Beyond mass spectrometry, the next step in proteomics. Sci. Adv..

[104] Ghezellou P., Garikapati V., Kazemi S.M., Strupat K., Ghassempour A., Spengler B. (2018). A perspective view of top-down proteomics in snake venom research. Rapid Commun. Mass Spectrom..

[105] Chang H.-C., Tsai T.-S., Tsai I.-H. (2013). Functional proteomic approach to discover geographic variations of king cobra venoms from Southeast Asia and China. J. Proteom..

[106] Petras D., Heiss P., Harrison R., Süssmuth R.D., Calvete J.J. (2016). Top-down venomics of the East African green mamba, *Dendroaspis angusticeps*, and the black mamba, *Dendroaspis polylepis*, highlight the complexity of their toxin arsenals. J. Proteom..

[107] Melani R.D., Nogueira F.C.S., Domont G.B. (2017). It is time for top-down venomics. J. Venom. Anim. Toxins Incl. Trop. Dis..

[108] Cristobal A., Marino F., Post H., Toorn H.W.P.V.D., Mohammed S., Heck A.J.R. (2017). Toward an Optimized Workflow for Middle-Down Proteomics. Anal. Chem..

[109] Wu C., Tran J.C., Zamdborg L., Durbin K.R., Li M., Ahlf D.R., Early B.P., Thomas P., Sweedler J., Kelleher N.L. (2012). A protease for ‘middle-down’ proteomics. Nat. Methods.

[110] Tasoulis T., Pukala T.L., Isbister G.K. (2020). Comments on Proteomic Investigations of Two Pakistani *Naja* Snake Venoms Species Unravel the Venom Complexity, Posttranslational Modifications, and Presence of Extracellular Vesicles. Toxins.

[111] Barua A., Mikheyev A.S. (2021). An ancient, conserved gene regulatory network led to the rise of oral venom systems. Proc. Natl. Acad. Sci. USA.

[112] Kerkkamp H.M.I., Kini R.M., Pospelov A.S., Vonk F.J., Henkel C.V., Richardson M.K. (2016). Snake Genome Sequencing: Results and Future Prospects. Toxins.

[113] Holding M.L., Margres M.J., Mason A.J., Parkinson C.L., Rokyta D.R. (2018). Evaluating the Performance of De Novo Assembly Methods for Venom-Gland Transcriptomics. Toxins.

[114] Tan C.H., Tan K.Y. (2021). De Novo Venom-Gland Transcriptomics of Spine-Bellied Sea Snake (*Hydrophis curtus*) from Penang, Malaysia—Next-Generation Sequencing, Functional Annotation and Toxinological Correlation. Toxins.

[115] Chong H.P., Tan K.Y., Tan N.H., Tan C.H. (2019). Exploring the Diversity and Novelty of Toxin Genes in *Naja sumatrana*, the Equatorial Spitting Cobra from Malaysia through De Novo Venom-Gland Transcriptomics. Toxins.

[116] Heather J.M., Chain B. (2015). The sequence of sequencers: The history of sequencing DNA. Genomics.

[117] Greenleaf W.J., Sidow A. (2014). The future of sequencing: Convergence of intelligent design and market Darwinism. Genome Biol..

[118] Jain M., Koren S., Miga K.H., Quick J., Rand A.C., Sasani T.A., Tyson J.R., Beggs A.D., Dilthey A.T., Fiddes I.T. (2018). Nanopore sequencing and assembly of a human genome with ultra-long reads. Nat. Biotechnol..

[119] Bleidorn C. (2015). Third generation sequencing: Technology and its potential impact on evolutionary biodiversity research. Syst. Biodivers..

[120] Suryamohan K., Krishnankutty S.P., Guillory J., Jevit M., Schröder M.S., Wu M., Kuriakose B., Mathew O.K., Perumal R.C., Koludarov I. (2020). The Indian cobra reference genome and transcriptome enables comprehensive identification of venom toxins. Nat. Genet..

[121] Weinstock G.M., Robinson G.E., Gibbs R.A., Weinstock G.M., Weinstock G.M., Robinson G.E., Worley K.C., Evans J.D., Maleszka R., Robertson H.M. (2006). Insights into social insects from the genome of the honeybee *Apis mellifera*. Nature.

[122] Vicoso B., Emerson J.J., Zektser Y., Mahajan S., Bachtrog D. (2013). Comparative Sex Chromosome Genomics in Snakes: Differentiation, Evolutionary Strata, and Lack of Global Dosage Compensation. PLoS Biol..

[123] Castoe T.A., de Koning A.P.J., Hall K.T., Card D.C., Schield D.R., Fujita M.K., Ruggiero R.P., Degner J.F., Daza J.M., Gu W. (2013). The Burmese python genome reveals the molecular basis for extreme adaptation in snakes. Proc. Natl. Acad. Sci. USA.

[124] Vonk F.J., Casewell N.R., Henkel C.V., Heimberg A.M., Jansen H.J., McCleary R.J.R., Kerkkamp H.M.E., Vos R.A., Guerreiro I., Calvete J.J. (2013). The king cobra genome reveals dynamic gene evolution and adaptation in the snake venom system. Proc. Natl. Acad. Sci. USA.

[125] Ullate-Agote A., Milinkovitch M.C., Tzika A.C. (2014). The genome sequence of the corn snake (*Pantherophis guttatus*), a valuable resource for EvoDevo studies in squamates. Int. J. Dev. Biol..

[126] Perry B.W., Card D.C., McGlothlin J.W., Pasquesi G.I.M., Adams R.H., Schield D.R., Hales N.R., Corbin A.B., Demuth J.P., Hoffmann F.G. (2018). Molecular Adaptations for Sensing and Securing Prey and Insight into Amniote Genome Diversity from the Garter Snake Genome. Genome Biol. Evol..

[127] Yin W., Wang Z.-J., Li Q., Lian J., Zhou Y., Lu B.-Z., Jin L.-J., Qiu P.-X., Zhang P., Zhu W.-B. (2016). Evolutionary trajectories of snake genes and genomes revealed by comparative analyses of five-pacer viper. Nat. Commun..

[128] Shibata H., Chijiwa T., Oda-Ueda N., Nakamura H., Yamaguchi K., Hattori S., Matsubara K., Matsuda Y., Yamashita A., Isomoto A. (2018). The habu genome reveals accelerated evolution of venom protein genes. Sci. Rep..

[129] Margres M.J., Rautsaw R.M., Strickland J.L., Mason A.J., Schramer T.D., Hofmann E.P., Stiers E., Ellsworth S.A., Nystrom G.S., Hogan M.P. (2021). The Tiger Rattlesnake genome reveals a complex genotype underlying a simple venom phenotype. Proc. Natl. Acad. Sci. USA.

[130] Li A., Wang J., Sun K., Wang S., Zhao X., Wang T., Xiong L., Xu W., Qiu L., Shang Y. (2021). Two Reference-Quality Sea Snake Genomes Reveal Their Divergent Evolution of Adaptive Traits and Venom Systems. Mol. Biol. Evol..

[131] Alföldi J., Di Palma F., Grabherr M., Williams C., Kong L., Mauceli E., Russell P., Lowe C.B., Glor R.E., Jaffe J.D. (2011). The genome of the green anole lizard and a comparative analysis with birds and mammals. Nature.

[132] Zhang G.J., Li C., Li Q.Y., Li B., Larkin D.M., Lee C., Storz J.F., Antunes A., Greenwold M.J., Meredith R.W. (2014). Comparative genomics reveals insights into avian genome evolution and adaptation. Science.

[133] (2004). International Chicken Genome Sequencing Consortium Sequence and comparative analysis of the chicken genome provide unique perspectives on vertebrate evolution. Nature.

[134] Majoros W.H., Pertea M., Salzberg S. (2004). TigrScan and GlimmerHMM: Two open source ab initio eukaryotic gene-finders. Bioinformatics.

[135] Pruitt K.D., Tatusova T., Brown G.R., Maglott D.R. (2011). NCBI Reference Sequences (RefSeq): Current status, new features and genome annotation policy. Nucleic Acids Res..

[136] Li H., Durbin R. (2010). Fast and accurate long-read alignment with Burrows–Wheeler transform. Bioinformatics.

[137] Langmead B., Salzberg S.L. (2012). Fast gapped-read alignment with Bowtie 2. Nat. Methods.

[138] Tatusov R.L., Fedorova N.D., Jackson J.D., Jacobs A.R., Kiryutin B., Koonin E.V., Krylov D.M., Mazumder R., Mekhedov S.L., Nikolskaya A.N. (2003). The COG database: An updated version includes eukaryotes. BMC Bioinform..

[139] Finn R.D., Mistry J., Tate J., Coggill P., Heger A., Pollington J.E., Gavin O.L., Gunasekaran P., Ceric G., Forslund S.K. (2009). The Pfam protein families database. Nucleic Acids Res..

[140] Palasuberniam P., Tan K.Y., Tan C.H. (2021). De novo venom gland transcriptomics of *Calliophis bivirgata flaviceps*: Uncovering the complexity of toxins from the Malayan blue coral snake. J. Venom. Anim. Toxins Incl. Trop. Dis..

[141] Aird S.D., Da Silva N.J., Qiu L., Villar-Briones A., Saddi V.A., Telles M.P.D.C., Grau M.L., Mikheyev A.S. (2017). Coralsnake Venomics: Analyses of Venom Gland Transcriptomes and Proteomes of Six Brazilian Taxa. Toxins.

[142] Margres M., McGivern J.J., Seavy M., Wray K.P., Facente J., Rokyta D.R. (2014). Contrasting Modes and Tempos of Venom Expression Evolution in Two Snake Species. Genetics.

[143] Rokyta D.R., Lemmon A.R., Margres M.J., Aronow K. (2012). The venom-gland transcriptome of the eastern diamondback rattlesnake (*Crotalus adamanteus*). BMC Genom..

[144] Spielman S.J., Wan S., Wilke C.O. (2016). A Comparison of One-Rate and Two-Rate Inference Frameworks for Site-Specific dN/dS Estimation. Genetics.

[145] Nakashima K., Nobuhisa I., Deshimaru M., Nakai M., Ogawa T., Shimohigashi Y., Fukumaki Y., Hattori M., Sakaki Y., Hattori S. (1995). Accelerated evolution in the protein-coding regions is universal in crotalinae snake venom gland phospholipase A2 isozyme genes. Proc. Natl. Acad. Sci. USA.

[146] Nakashima K., Ogawa T., Oda N., Hattori M., Sakaki Y., Kihara H., Ohno M. (1993). Accelerated evolution of *Trimeresurus flavoviridis* venom gland phospholipase A2 isozymes. Proc. Natl. Acad. Sci. USA.

[147] Casewell N.R. (2012). On the ancestral recruitment of metalloproteinases into the venom of snakes. Toxicon.

[148] Castoe T.A., Hall K.T., Mboulas M.L.G., Gu W., de Koning A.J., Fox S.E., Poole A.W., Vemulapalli V., Daza J.M., Mockler T. (2011). Discovery of Highly Divergent Repeat Landscapes in Snake Genomes Using High-Throughput Sequencing. Genome Biol. Evol..

[149] Aird S.D. (2001). Ophidian envenomation strategies and the role of purines. Toxicon.

[150] Li M., Fry B.G., Kini R.M. (2005). Putting the Brakes on Snake Venom Evolution: The Unique Molecular Evolutionary Patterns of *Aipysurus eydouxii* (Marbled Sea Snake) Phospholipase A2 Toxins. Mol. Biol. Evol..

[151] Tekaia F. (2016). Inferring Orthologs: Open Questions and Perspectives. Genom. Insights.

[152] Vonk F.J., Admiraal J.F., Jackson K., Reshef R., de Bakker M., Vanderschoot K., Berge I.V.D., Van Atten M., Burgerhout E., Beck A. (2008). Evolutionary origin and development of snake fangs. Nature.

[153] Dowell N.L., Giorgianni M.W., Kassner V.A., Selegue J.E., Sanchez E.E., Carroll S.B. (2016). The Deep Origin and Recent Loss of Venom Toxin Genes in Rattlesnakes. Curr. Biol..

[154] Almeida D.D., Viala V.L., Nachtigall P.G., Broe M., Gibbs H.L., Serrano S.M.D.T., Moura-Da-Silva A.M., Ho P.L., Nishiyama M.Y., Junqueira-De-Azevedo I.L.M. (2021). Tracking the recruitment and evolution of snake toxins using the evolutionary context provided by the *Bothrops jararaca* genome. Proc. Natl. Acad. Sci. USA.

[155] Telford M.J., Copley R.R. (2011). Improving animal phylogenies with genomic data. Trends Genet..

[156] Laustsen A.H., Maria Gutiérrez J., Knudsen C., Johansen K.H., Bermúdez-Méndez E., Cerni F.A., Jürgensen J.A., Ledsgaard L., Martos-Esteban A., Øhlenschlæger M. (2018). Pros and cons of different therapeutic antibody formats for recombinant antivenom development. Toxicon.

[157] Tan C.H., Lingam T.M.C., Tan K.Y. (2022). Varespladib (LY315920) rescued mice from fatal neurotoxicity caused by venoms of five major Asiatic kraits (*Bungarus* spp.) in an experimental envenoming and rescue model. Acta Trop.

[158] Salvador G.H.M., Gomes A.A.S., Bryan-Quiros W., Fernandez J., Lewin M.R., Gutierrez J.M., Lomonte B., Fontes M.R.M. (2019). Structural basis for phospholipase A2-like toxin inhibition by the synthetic compound Varespladib (LY315920). Sci Rep.

[159] Rockman M.V., Kruglyak L. (2006). Genetics of global gene expression. Nat. Rev. Genet..

[160] Conesa A., Madrigal P., Tarazona S., Gomez-Cabrero D., Cervera A., McPherson A., Szcześniak M.W., Gaffney D.J., Elo L.L., Zhang X. (2016). A survey of best practices for RNA-seq data analysis. Genome Biol..

[161] Oshlack A., Robinson M.D., Young M.D. (2010). From RNA-seq reads to differential expression results. Genome Biol..

[162] Op den Brouw B., Coimbra F.C.P., Bourke L.A., Huynh T.M., Vlecken D.H.W., Ghezellou P., Visser J.C., Dobson J.S., Fernandez-Rojo M.A., Ikonomopoulou M.P. (2021). Extensive Variation in the Activities of Pseudocerastes and Eristicophis Viper Venoms Suggests Divergent Envenoming Strategies Are Used for Prey Capture. Toxins.

[163] Powell D.W., Weaver C.M., Jennings J.L., McAfee K.J., He Y., Weil P.A., Link A.J. (2004). Cluster Analysis of Mass Spectrometry Data Reveals a Novel Component of SAGA. Mol. Cell. Biol..

[164] Old W., Meyer-Arendt K., Aveline-Wolf L., Pierce K.G., Mendoza A., Sevinsky J.R., Resing K.A., Ahn N.G. (2005). Comparison of Label-free Methods for Quantifying Human Proteins by Shotgun Proteomics. Mol. Cell. Proteom..

[165] Heck M., Neely B. (2020). Proteomics in non-model organisms: A new analytical frontier. J. Proteome Res..

